# Single-molecule visualization of human RECQ5 interactions with single-stranded DNA recombination intermediates

**DOI:** 10.1093/nar/gkaa1184

**Published:** 2020-12-17

**Authors:** Chaoyou Xue, Lucia Molnarova, Justin B Steinfeld, Weixing Zhao, Chujian Ma, Mario Spirek, Kyle Kaniecki, Youngho Kwon, Ondrej Beláň, Katerina Krejci, Simon J Boulton, Patrick Sung, Eric C Greene, Lumir Krejci

**Affiliations:** Department of Biochemistry & Molecular Biophysics, Columbia University, New York, NY 10032, USA; Department of Biology, Masaryk University, Brno 62500, Czech Republic; Department of Biochemistry & Molecular Biophysics, Columbia University, New York, NY 10032, USA; Department of Biochemistry and Structural Biology, University of Texas Health Science Center at San Antonio, TX 78229, USA; Department of Biochemistry & Molecular Biophysics, Columbia University, New York, NY 10032, USA; Department of Biology, Masaryk University, Brno 62500, Czech Republic; Department of Biochemistry & Molecular Biophysics, Columbia University, New York, NY 10032, USA; Department of Biochemistry and Structural Biology, University of Texas Health Science Center at San Antonio, TX 78229, USA; DSB Repair Metabolism Lab, The Francis Crick Institute, Midland Road, London NW1 1AT, UK; Department of Biology, Masaryk University, Brno 62500, Czech Republic; International Clinical Research Center, St. Anne's University Hospital Brno, Brno 65691, Czech Republic; DSB Repair Metabolism Lab, The Francis Crick Institute, Midland Road, London NW1 1AT, UK; Department of Biochemistry and Structural Biology, University of Texas Health Science Center at San Antonio, TX 78229, USA; Department of Biochemistry & Molecular Biophysics, Columbia University, New York, NY 10032, USA; Department of Biology, Masaryk University, Brno 62500, Czech Republic; International Clinical Research Center, St. Anne's University Hospital Brno, Brno 65691, Czech Republic; National Centre for Biomolecular Research, Masaryk, Brno 62500, Czech Republic

## Abstract

RECQ5 is one of five RecQ helicases found in humans and is thought to participate in homologous DNA recombination by acting as a negative regulator of the recombinase protein RAD51. Here, we use kinetic and single molecule imaging methods to monitor RECQ5 behavior on various nucleoprotein complexes. Our data demonstrate that RECQ5 can act as an ATP-dependent single-stranded DNA (ssDNA) motor protein and can translocate on ssDNA that is bound by replication protein A (RPA). RECQ5 can also translocate on RAD51-coated ssDNA and readily dismantles RAD51–ssDNA filaments. RECQ5 interacts with RAD51 through protein–protein contacts, and disruption of this interface through a RECQ5–F666A mutation reduces translocation velocity by ∼50%. However, RECQ5 readily removes the ATP hydrolysis-deficient mutant RAD51–K133R from ssDNA, suggesting that filament disruption is not coupled to the RAD51 ATP hydrolysis cycle. RECQ5 also readily removes RAD51–I287T, a RAD51 mutant with enhanced ssDNA-binding activity, from ssDNA. Surprisingly, RECQ5 can bind to double-stranded DNA (dsDNA), but it is unable to translocate. Similarly, RECQ5 cannot dismantle RAD51-bound heteroduplex joint molecules. Our results suggest that the roles of RECQ5 in genome maintenance may be regulated in part at the level of substrate specificity.

## INTRODUCTION

RecQ helicases constitute a unique subgroup of the SF2 (super-family 2) helicases and they play essential roles in the maintenance of genome integrity (1–8). Humans possess five RecQ homologs, namely WRN, BLM, RECQ1, RECQ4 and RECQ5 (1–7,9). Mutations in BLM, WRN and RECQ4 cause Bloom, Werner and Rothmund-Thompson syndromes, respectively, which are associated with profound developmental abnormalities and increased cancer risk, and the latter two syndromes are also characterized by premature ageing (1–7). The average Bloom syndrome patient lifespan is only 27 years and cancer is the leading cause of death (10–13). Cells from patients with Bloom Syndrome (BS) are marked by DNA damage hypersensitivity, elevated genome instability, and a ∼10-fold increase in sister chromatid exchanges (SCEs) (10–13). The SCE phenotype of BLM deficient cells reflects a failure to suppress excessive crossover formation during homologous recombination (10–13). Efforts to more fully understand the roles of human RECQ helicases in the maintenance of genome integrity are confounded by the partial functional overlap of these proteins (1–8).

The repair of double-strand breaks (DSBs) and protection of stalled replication forks by HR is essential to maintain genome integrity. The RAD51 protein bound to ssDNA performs key functions in these processes, including the homology search, DNA strand exchange and ssDNA protection (14–16). However, untimely formation of RAD51 filaments can imply the presence of recombination intermediates that cannot be repaired properly, which can lead to gross chromosomal rearrangements and increased cancer risk. To help avoid these problems cells employ proteins known as antirecombinases to dismantle toxic Rad51 filaments (1–8). RECQ5 (also called RECQL5) is a tumor suppressor protein and antirecombinase that has been implicated as a possible breast cancer susceptibility gene (17,18), but its precise functions have been difficult to ascertain, in part because there is no known *RECQ5*-associated syndrome (1,7). However, a population-based study of all five human *RECQ* helicase genes failed to identify any *RECQ5* loss of function mutations, suggesting that the lack of a *RECQ5*-associated syndrome may be due to embryonic lethality (19). In human cells, RECQ5 depletion causes genomic instability (20), and *RECQ5*knockout mice display chromosomal instability, elevated RAD51 foci and cancer predisposition (21). Elevated RECQ5 expression is linked to urothelial bladder carcinoma, which affects ∼400 000 people annually (22), moreover, the *RECQ5* gene is frequently amplified in human cancers and even moderate overexpression of RECQ5 in human cell lines can lead to dysregulation of homologous recombination (23). RECQ5 is thought to regulate homologous recombination by disrupting RAD51–ssDNA (3,21,24,25), RECQ5 also associates with RNA pol I & II (20,26–28), participates in transcription-coupled repair and R-loop disruption (28), resolution of replication/transcription conflicts (29), and acts synergistically with WRN and BLM to maintain genome integrity (22). In addition, RECQ5 associates with common fragile sites (CFS) during mitosis to remove the RAD51 nucleoprotein filaments stabilizing stalled replication forks, thereby promoting CFS cleavage by MUS81–EME1 (30). These findings underscore the importance of understanding how RECQ5 acts on RAD51 nucleoprotein filaments.

The RecQ helicases share a common structural helicase core domain, but RECQ5 is distinct from WRN and BLM in that it lacks the otherwise highly conserved helicase and RNase D-like C-terminal (HRDC) domain (1,4,5,31). Studies with *E. coli* RecQ and the yeast BLM homolog Sgs1 suggest that the HRDC domain is not required for ATP hydrolysis or helicase activity, but it promotes stable DNA binding (4,32,33). Consequently, the BLM and WRN HRDC domain play important roles in recruitment to DNA damage (1,4,5,31). RECQ5 also lacks an intact RecQ C-terminal (RQC) domain, which includes the winged helix (WD) domain presumed to be involved in binding dsDNA (1,4,5). These important structural differences may reflect important functional differences in RECQ5 compared to other RecQ family helicases. RECQ5 can disrupt RAD51–ssDNA filaments and in doing so acts as a negative regulator of RAD51 strand invasion activity (3,21). Interestingly, RECQ5 can dismantle active ATP-bound forms of the RAD51–ssDNA filament, whereas BLM can only dismantle the inactive ADP-bound form of RAD51 (3,25,34,35). This important difference suggests the possibility that RECQ5 may play a more prominent role than BLM in regulating RAD51 filament disassembly (3). The ability to remove RAD51 from ssDNA is thought to promote the synthesis dependent strand annealing (SDSA) pathway of homologous recombination (HR) (3), and has been linked to the participation of RECQ5 in processing stalled replication forks at common fragile sites (30).

Despite its clear links to genome integrity, the precise functions of RECQ5 have been difficult to fully define because it has been implicated in numerous processes, has numerous interacting partners, and may have partial functional redundancy with the other RECQ helicases (1–7). To help further delineate the roles of RecQ helicases in genome maintenance, here we developed a simple and rapid experimental system enabling real-time monitoring of RECQ5 antirecombinase activity on various nucleoprotein complexes by single-molecule method and biolayer interferometry. Our data reveal that RECQ5 is a robust ssDNA motor protein capable of rapid and processive translocation on ssDNA bound by RPA, RAD51 or DMC1 while removing these proteins from the ssDNA. We also show that RECQ5 acts as a multimeric complex that can translocate at a velocity of ∼60–80 nucleotides per second for average distances of ∼10 000 nucleotides on a variety of protein-bound ssDNA intermediates. However, we find that RECQ5 is unable to unwind long dsDNA substrates. Moreover, unlike budding yeast Srs2 (47), RECQ5 removes the ATP hydrolysis-deficient mutant RAD51–K133A and K133R from ssDNA, suggesting that RAD51 filament disruption is not coupled to the RAD51 ATP binding or hydrolysis. Furthermore, we show that RECQ5 is readily recruited to RAD51-bound heteroduplex DNA joints, but it is unable to dismantle these intermediates. Our findings provide new quantitative insights into the function of the RECQ5 ssDNA motor activity and suggest potential roles in genome integrity.

## MATERIALS AND METHODS

### Proteins

The GFP–RECQ5 expression plasmid was constructed by inserting the GFP sequence into the plasmid pTXB1–RECQ5 (25). The sequences encoding GFP with monomerizing A206K mutation were amplified by PCR using pET11–GFP–RPA as a template. The primers used for amplification contained extra His-tag and Alanines resulting in NdeI–8xHis–GFP–8xAlanine–NdeI construct, which was then inserted into the pTXB1–RECQ5 plasmid digested by NdeI. The GFP–RECQ5 expression plasmid was verified by sequencing.

Human RECQ5, RECQ5–F666A (FA), GFP–RECQ5 were purified similarly to the previously described protocol, with few modifications (25). Expression plasmid pTXB1–RECQ5 was introduced into the *E. coli* ARCTIC RIL strain (Agilent). The culture was grown to an OD 0.7 at 37°C in 2 × TY media supplemented with ampicillin (100 mg/l), shifted to 11°C and then induced over night with 1 mM IPTG. The bacterial pellet was resuspended in cell breakage buffer (CBB) (50 mM Tris–HCl [pH 7.5], 10% sucrose, 0.5 mM EDTA, 500 mM KCl, 0.01% NP-40, cocktail of protease inhibitors (Sigma-Aldrich, Cat. No. 11697498001) and 1 mM PMSF, sonicated and centrifuged at 100 000 × g for 1 h. The clarified extract was mixed with chitin beads (New England BioLabs) preequilibrated in T buffer (25 mM Tris–HCl [pH 7.5], 10% glycerol, 0.5 mM EDTA and 0.01% NP-40) supplemented with 500 mM KCl. To release the RECQ5 protein from the intein tag, T buffer with 500 mM KCl and 50 mM DTT was added to the beads, and incubated O/N on a rotary shaker at 4°C. Cleaved RECQ5 protein was eluted with buffer T containing 150 mM KCl and 1 mM DTT. RECQ5 peak protein fractions were pooled and loaded onto a hydroxyapatite column (Sigma-Aldrich) equilibrated with T buffer containing 150 mM KCl and 1 mM DTT. RECQ5 protein was eluted by linear gradient of 0–1000 mM KH_2_PO_4_ gradient in T buffer. Pooled peak fractions were loaded on Mono S column (GE Healthcare) equilibrated with T buffer with 150 mM KCl and 1 mM DTT. RECQ5 protein was eluted with 0–800 mM KCl gradient in T buffer. Peak RECQ5 fractions were pooled, concentrated using Vivaspin Centrifugal Concentrator (30,000 MWCO polyethersulfone [PES]) and stored in small aliquots at −80°C.

For, BLI measurements, Human wild-type (wt) RAD51, RAD51–K133R (KR), RAD51–K133A (KA) and RAD51–I287T (IT) were expressed in *E. coli* and purified as previously described (36). Briefly, the proteins were purified by ammonium sulphate precipitation (0.242 mg/ml) followed by chromatography on Q Sepharose Fast Flow column (GE Healthcare), hydroxyapatite column (Sigma-Aldrich) and Mono Q column (GE Healthcare). Peak fractions were pooled and concentrated using Vivaspin Centrifugal Concentrator (30 000 MWCO PES), aliquoted and stored at −80°C. DMC1 was purified similar to the previously described protocol, with several modifications (37). The expression plasmid pET15b–hDmc1 was introduced into BLR (DE3) *E. coli* strain. The culture was grown to an OD 0.7 at 37°C in 2 × TY media supplemented with ampicillin (100 mg/L), and protein expression was induced by adding 1 mM IPTG. Cells were harvested 3 h after IPTG induction by centrifugation and lysed by sonication in buffer containing 50 mM Tris–HCl [pH 7.5], 10% sucrose, 0.5 mM EDTA, 600 mM KCl, 0.01% NP-40, 10 mM 2-mercaptoethanol, cocktail of protease inhibitors (Sigma-Aldrich, Cat. No. 11697498001), and 1 mM PMSF. The cell lysate was centrifuged at 100 000 × g for 1 h and the supernatant was mixed with Ni-NTA agarose beads (Sigma) pre-equilibrated in T buffer supplemented with 200 mM KCl and 10 mM 2-mercaptoethanol for 1 h. DMC1 protein was eluted by step gradient of imidazole (50, 150, 300, 500, 1000 mM) in buffer T with 50 mM KCl. The fractions containing DMC1 protein were pooled and applied onto hydroxyapatite (Sigma-Aldrich) column equilibrated with T buffer containing 100 mM KCl and 10 mM 2-mercaptoethanol. The DMC1 protein was eluted by linear gradient of 100–1000 mM KH_2_PO_4_ gradient in T buffer. The DMC1 peak fractions were then loaded on Mono Q column (GE Healthcare) pre-equilibrated with T buffer containing 100 mM KCl and 10 mM 2-mercaptoethanol and eluted by 170–850 mM KCl gradient in T buffer. The peak DMC1 fractions were applied onto a Superdex 200 (GE Healthcare) gel filtration column equilibrated with T supplemented with 150 mM KCl and 10 mM 2-mercaptoethanol. The protein was eluted using the same buffer, and fractions containing DMC1 were polled, concentrated using Vivaspin Centrifugal Concentrator (30 000 MWCO PES) and stored at −80°C.

For DNA curtain measurements, human RAD51, RAD51–K133R, RAD51–I287T were purified as previously described (38) with minor modifications. Briefly, RAD51 was expressed using pET–SUMO–RAD51 construct in Rossetta (DE3) pLys. Six liters of *E. coli* cells in LB were grown at 37°C, induced 4 h at 37°C with 0.2 mM IPTG when OD_600_ reached 0.5, and then harvested by centrifugation. The cells were resuspended in 50 ml cell lysis buffer (50 mM Tris (7.5), 10% sucrose, 1 M NaCl, 10 mM EDTA, 1 mM tris(2-carboxyethyl)phosphine hydrochloride [TCEP]), 0.1% Triton, protease inhibitor cocktail (Roche, Cat. No. 05892988001)) and lysed by sonication. The extract was centrifuged for 1 h at 40 000 rpm at 4°C using a Ti-45 rotor. The supernatant was precipitated with 0.24 mg/ml ammonium sulfate for 1 h. The pellet was recovered by centrifugation at 10 000 rpm at 4°C for 10 min. Then, the pellet was dissolved in His loading buffer (25 mM Tris [pH 7.5], 10% glycerol, 1 M NaCl, 1 mM TCEP, 15 mM imidazole) for 1 h. The resuspension was cleared by centrifugation at 15 000 rpm at 4°C for 10 min. The protein solution was purified through a Ni-NTA column (Roche) and further treated with SUMO protease to cleave off the His6-SUMO tag while dialyzing into dialysis buffer (25 mM Tris [pH 7.5], 10% glycerol, 1 M NaCl, 1 mM TCEP at 4°C for overnight. RAD51 was further purified by passaging through a Ni-NTA column to remove the cleaved His6-SUMO tag. RAD51 protein was concentrated and stored at −80°C. Human DMC1 was purified in SF9 insect cell. pFastbac-His-DMC1 was introduced into *E. coli* strain DH10Bac for bacmid generation. The bacmids were used to transfect SF9 insect cells to generate recombinant baculoviruses. After amplification in SF9 cells, 10 ml DMC1 viruses the viruses were used to infect 600 ml Hi5 insect cells. After a 44-h incubation at 27°C, cells were harvested by centrifugation, frozen in liquid nitrogen, and stored at −80°C. All the purification steps were carried out at 0 to 4°C. To prepare extract, 10 g of cell paste was suspended in 50 ml of cell breakage buffer A (25 mM Tris–HCl [pH 7.5], 10% glycerol, 0.5 mM EDTA, 100 mM KCl, 0.01% Igepal and 1 mM DTT), and the following protease inhibitors: aprotinin, chymostatin, leupeptin and pepstatin A at 3 mg/ml each, and 1 mM PMSF) for sonication. After centrifugation (100 000 × g for 90 min), the clarified lysate was loaded on 20 ml Q fast flow sepharose (Amersham Pharmacia Biotech) column and then the column was developed with 100–600 mM KCl gradient (200 ml) in buffer B buffer A (25 mM Tris–HCl [pH 7.5], 10% glycerol, 0.5 mM EDTA, 0.01% Igepal and 1 mM DTT). The fractions containing DMC1 were pooled together for incubation with 3 ml Ni^2+^ NTA-agarose (Qiagen) for 1 h. The matrix was poured into a column, washed with 50 ml buffer B with 1000 mM KCl and then with 10 ml each of 30 and 50 mM imidazole in buffer A before being eluted with 15 ml of 200 mM imidazole in buffer A. The 200 mM imidazole eluate were further fractionated in a 1 ml Mono Q column (Amersham Pharmacia Biotech), using a 15 ml gradient of 200–600 mM KCl in buffer B. The fractions containing DMC1 were diluted to 50 mM KCl and loaded on a 1 ml Mono S column for being devloped with 10 ml gradient of 100–500 mM KCl in buffer B. DMC1 containing fractions were pooled and concentrated in a Centricon-30 concentrator (Amicon) before small aliquots and storage at −80°C. Note that the His6 tag was not removed from the N-terminus of DMC1 prior to use.

### Atomic force microscopy

Samples were deposited on freshly cleaved mica (muscovite V-1 quality, from Electron Microscopy Science) mounted on microscope slides (Marienfeld). After 2 min, the sample drop was rinsed with MilliQ water and dried with filtered air. Atomic force microscopy was done with NanoWizard 3 (JPK Instruments) mounted on the fluorescence microscope (Leica). Air-dried samples were scanned in intermittent contact mode (air). Super Sharp Silicon (SSS-MCHR-50) scanning probes by Nanosensors were used. Volume analysis was acquired with SFMetricsV4c1 software. Calibration of protein size was done using RPA (116 kDa) which had a mean volume of 130 nm^3^.

### ATP hydrolysis assays with RECQ5 and GFP–RECQ5

Comparison of unlabeled RECQ5 and GFP–RECQ5 ATP hydrolysis activities was performed in RECQ5 buffer (20 mM Tris–HCl [pH 7.5], 1 mM MgCl_2_, 5 mM CaCl_2_, 50 mM KCl, 2 mM ATP, 1 mM DTT, 0.2 mg/ml BSA) at 37°C. All reactions contained either M13 ssDNA (2 μM nucleotides; NEB, Cat. No. N4040S) or pUC19 dsDNA (2 μM nucleotides; NEB, Cat No. N3041A). Reactions were initiated by the addition of 10 nM RECQ5 or GFP–RECQ5, as indicated. Aliquots were removed at the indicated time points and quenched by addition of 50 mM EDTA. The quenched reactions were quantified by the ATPase/GTPase Activity Assay Kit as per the manufacturer's instructions (Sigma, Cat. No. MAK113).

ATP hydrolysis assays testing for the effects of RPA and RAD51 were also performed in RECQ5 buffer at 37°C. RPA or RAD51 with indicated concentrations were incubated with M13 ssDNA (2 μM nucleotides) for 10 min at 37°C before the addition of 10 nM RECQ5, RECQ5–F666A, or GFP–RECQ5. Aliquots were removed at the indicated time points and quenched by addition of 50 mM EDTA and were quantified as described above.

### D-loop disruption assays

To form the nucleoprotein filament, RAD51 (2 μM) or DMC1 (2 μM) was incubated with 40 nM 5′-fluorescein-labeled 90-mer oligonucleotide (5′-AAA TCA ATC TAA AGT ATA TAT GAG TAA ACT TGG TCT GAC AGT TAC CAA TGC TTA ATC AGT GAG GCA CCT ATC TCA GCG ATC TGT CTA TTT-3′) in D-loop buffer (25 mM Tris–HCl [pH 7.5], 50 mM KCl, 1 mM DTT, 2 mM MgCl_2_, 2 mM CaCl_2,_100 μg/ml BSA, 20 mM creatine phosphate and 20 μg/ml creatine kinase) for 10 min at 37°C. RECQ5 (in indicated concentrations) and RPA (300 nM) were added to the nucleoprotein filament and incubated for 5 min, followed by the addition of HOP2-MND1 (300 nM) for 2 min. D-loop formation was initiated by addition of pBluescript SK(–) (500 ng) to bring the final reaction volume to 10 μl and incubated for another 10 min at 37°C. The reactions were deproteinized by addition of 10 μg proteinase K and 0.1% SDS and incubated for 10 min at 37°C and analyzed by electrophoresis in a 0.9% agarose gel in 1× TAE (90 V, 30 min). Gels were imaged on a FLA-9000 scanner (Fujifilm) and quantified with Multi Gauge V3.2 (Fujifilm).

### Biolayer Interferometry measurements (BLI)

BLI experiments were performed using a single-channel BLItz instrument (ForteBio) in Advanced Kinetics mode at room temperature and with shaking at 2,200 rpm. For RAD51 filament disruption experiments, prior to the measurements the streptavidin biosensors (SAX, ForteBio, Cat No. 18-0037) were pre-hydrated by incubation with BLI buffer (25 mM Tris–HCl [pH 7.5], 50 mM KCl, 2 mM MgCl_2_, 200 μg/mL BSA, and 0.05% Tween 20) for 10 min. BLI experiments consisted of 5 steps performed in BLI buffer supplemented with 2 mM ATP: (i) recording initial baseline with BLI buffer for 30 s; (ii) loading 5′ biotinylated dT43-mer (40 nM) for 120 s; (iii) washing with BLI buffer for 30 s; (iv) association step with 2 μM RAD51 protein for 240 s to form the presynaptic filament, (v) dissociation step with 40 nM RECQ5 and 2 μM BRC3 peptide for another 240 s. Please note that all BLI experiments looking at RECQ5-mediated removal of RAD51 from ssDNA include the BRC3 peptide to prevent RAD51 from rebinding to the ssDNA once it dissociates. For all experiments monitoring RECQ5 translocase activity, controls were performed with BRC3 peptide only (minus RECQ5). The data from the time period where BRC3 without RECQ5 was added to the RAD51 filament, were normalized to the starting point. The change in signal was then plotted as a function of time for each nucleoprotein filament.

For protein-protein interaction measurements, protein A biosensors (ForteBio, Cat No. 18-0028) were incubated with BLI buffer for 10 min prior to use. These protein–protein interaction BLI experiments consisted of three steps: (i) recording initial baseline with BLI buffer; (ii) loading of 0.5 μg/ml rabbit polyclonal RECQ5 antibody pre-incubated with 1.5 μM RECQ5 for 10 min; (iii) association step with 2.5 μM RAD51, DMC1 or RPA protein. The binding affinity of the proteins was measured as an increase in the thickness of the biomolecule layer in nanometers (nm). All BLI measurements were performed in triplicate.

As a control to test for RAD51 interactions with BRC3, BLI experiments using anti-GST biosensors (ForteBio, Cat No. 18-5096) and immobilized GST–BRC3. Biosensors were coated by 1 μM GST–BRC3 for 120 s, washed and subsequently mixed with 1 μM Rad51 variants for 120 s (association phase). The dissociation phase was initiated by flushing the biosensor to BLI buffer for 120 s. Shaker speed was set to 1800 rpm.

### Single molecule data collection

All single molecule experiments were conducted with a prism-type total internal reflection fluorescence (TIRF) microscope (Nikon) equipped with a 488-nm laser (Coherent Sapphire, 200 mW), a 561-nm laser (Coherent Sapphire, 200 mW), and two Andor iXon EMCCD cameras (39,40). The microscope was equipped with a 60x Nikon objective lens (N.A. 1.2), yielding an optical resolution limit of 254 nm at a wavelength of 500 nm. Flowcells and ssDNA curtains were prepared as previously described (39,40). In brief, lipid bilayers were prepared with 91.5% DOPC (1,2-dioleoyl-sn-glycero-3-phosphocholine), 0.5% biotinylated-PE (1,2-dioleoyl-sn-glycero-3-phosphoethanolamine-*N*-(biotinyl) (sodium salt)), and 8% mPEG 2000-DOPE (1,2-distearoyl-sn-glycero-3-phosphoethanolamine-*N*-[methoxy(polyethylene glycol)-2000] (ammonium salt)). All lipids were purchased from Avanti Polar Lipids. The ssDNA substrate was generated using rolling circle replication with a biotinylated primer, a circular M13 ssDNA template, and phi29 DNA polymerase, as described (39,40). The biotinylated ssDNA was injected into the sample chamber and attached to the bilayer through a biotin-streptavidin linkage. The flow cell was then attached to a microfluidic system and sample delivery was controlled using a syringe pump (KD Scientific) (39,40). For all two-color images, we used a custom-built shuttering system to avoid signal bleed-through during image acquisition. With this system, images from the green (GFP) and the red (mCherry) channels are recorded independently, these recordings are offset by 100 ms such that when one camera records the red channel image, the green laser is shuttered off, and vice versa (39,40).

### Recombinase filament assembly and disassembly kinetics

The rates of spontaneous recombinase filament assembly and disassembly, in the absence of RECQ5, were measured using ssDNA curtains assays, as follows. Biotinylated ssDNA was aligned at the barriers by application of flow in BSA buffer (40 mM Tris–HCl [pH 8.0], 1 mM MgCl_2_, 1 mM DTT, 0.2 mg/ml BSA) at 37°C with a flow rate of 1 ml/min. Secondary structure was reduced with a 500 μl 7 M urea, immediately followed by 5–10 ml of BSA buffer containing 100 pM unlabeled RPA, RPA–GFP or mCherry–RPA, as indicated. RPA was flushed out with either RAD51 HR buffer (30 mM Tris–acetate [pH 7.5], 1 mM MgCl_2_, 5 mM CaCl_2_, 100 mM KCl, 2 mM ATP, 1 mM DTT, 0.2 mg/ml BSA) or DMC1 HR buffer (40 mM Tris [pH 7.5], 2 mM MgCl_2_, 1.5 mM CaCl_2_, 100 mM KCl, 2.5 mM ATP, 1 mM DTT, 0.2 mg/ml BSA) at 1 ml/min for 2 min. Then, either RAD51 (2 μM), RAD51–K133R (2 μM), RAD51–I287T (2 μM) or DMC1 (2 μM) in a 50 μl loop was injected into the flow cell, buffer flow was terminated when protein reached the flow cell, and the reactions were incubated at 37°C for 20 min. The loss of RPA fluorescence signal was monitored to verify recombinase filament assembly. The time-increase decrease in the normalized RPA–GFP fluorescence intensity (integrated over entire ssDNA molecules) reflects the disassembly of RPA–GFP, which is used for the calculation of RAD51 assembly (39,40). The resulting intensity curves were fitted to a simple exponential decay function: *I* = *A* × e^–k*t^, where *I* is the normalized fluorescence intensity, *k* is the observed disassembly rate of RPA, which is equal to RAD51 assembly rate.

For disassembly reactions, presynaptic complexes were formed as described above. Disassembly was then initiated by resuming buffer flow with HR buffer lacking both CaCl_2_ and ATP, which contained 0.5 nM free RPA–GFP. The rebinding of RPA–GFP to the exposed ssDNA was monitored to verify recombinase filament disassembly. The intensity curves of RPA–GFP were fitted to an exponential recovery function: *I* = *A* × (1 – e^–k*t^), where *k* is the observed RPA association rate which is equal to RAD51 dissociation rate.

### RECQ5 translocation assays and data analysis

All RECQ5 measurements were conducted at 37°C in reaction buffer (20 mM Tris–HCl [pH 7.5], 1 mM MgCl_2_, 5 mM CaCl_2_, 50 mM KCl, 2 mM ATP, 1 mM DTT, 0.2 mg/ml BSA) supplemented with RPA (unlabeled, GFP-tagged, or mCherry-tagged, as indicated). 50 μl samples containing the indicated GFP–RECQ5 concentrations plus 0.5 nM RPA–mCherry, RPA–GFP or unlabeled RPA (as indicated) were injected into the flow cell at a rate of 0.2 ml/min, and RECQ5 activity was observed under constant buffer flow in reaction buffer (plus 0.5 nM RPA–mCherry, RPA–GFP or unlabeled RPA, as indicated) for a total period of 20–25 minutes. All data were collected as previously described for Srs2 (40–42). In brief, images with captured at an acquisition rate of 1 frame per 10 s with a 100-ms integration time, and the laser was shuttered between each acquired image to minimize photo-bleaching. Raw TIFF images were imported as image stacks into ImageJ, and kymographs were generated from the image stacks by defining a 1-pixel wide region of interest (ROI) along the long-axis of the individual ssDNA molecules. RECQ5 translocation velocity was calculated from the kymographs by manually measuring the distance travelled as a function of time. The velocities were then plotted in 15 nt/s bins and the resulting histograms were fit to a Gaussian distribution using Prism 7 (Graphpad Software, Inc.). Reported velocities represent the mean ± the standard deviation generated from the Gaussian fits. For RECQ5 processivity, the distance a molecule travelled was calculated in pixels, the distance values were then changed to nucleotides using a conversion factor of 725 nt/pixel, and the resulting data were used to generate survival plots, as described (40–42). The survival plots were fit as single exponential decay curves, and the reported processivity values corresponds to the half-life obtained from these curves. Error bars were generated by bootstrapping using a custom python script.

## RESULTS

### RECQ5 is a molecular motor that can translocate on RPA-coated ssDNA

Single stranded DNA (ssDNA) is an intermediate in many aspects of DNA replication and repair. However, naked ssDNA is unlikely to exist in physiological settings, instead it quickly becomes sequestered by the abundant heterotrimeric ssDNA-binding protein complex RPA (43–45). For instance, during HR, RPA-coated ssDNA is present after DSB end resection and is a necessary precursor for the assembly of subsequent HR intermediates, and RPA–ssDNA is also a core component of the eukaryotic replisome to protect ssDNA at the fork (40–42). Given this importance, we have established RPA-coated ssDNA curtain assays for visualizing the behaviors of helicases on ssDNA intermediates in real-time (see below) (39,40). In brief, long ssDNA substrates (≥50 kilonucleotides; knt) are generated by rolling circle replication using a 5′ biotinylated primer (39,40). The resulting 5′ biotinylated ssDNA is tethered to a supported lipid bilayer on the surface of a microfluidic sample chamber through a biotin–streptavidin linkage (39,40). The 5′ ends of the ssDNA are then aligned at nanofabricated chromium (Cr) barriers to lipid diffusion and the downstream ends of the ssDNA are attached to Cr pedestals, which are deposited onto the fused silica by electron beam lithography (39,40). The addition of GFP- or mCherry-tagged RPA allows the ssDNA to be extended by hydrodynamic force, and also provides means of visualizing the ssDNA by total internal reflection fluorescence microscopy (TIRFM) (39,40).

**Figure 1.**
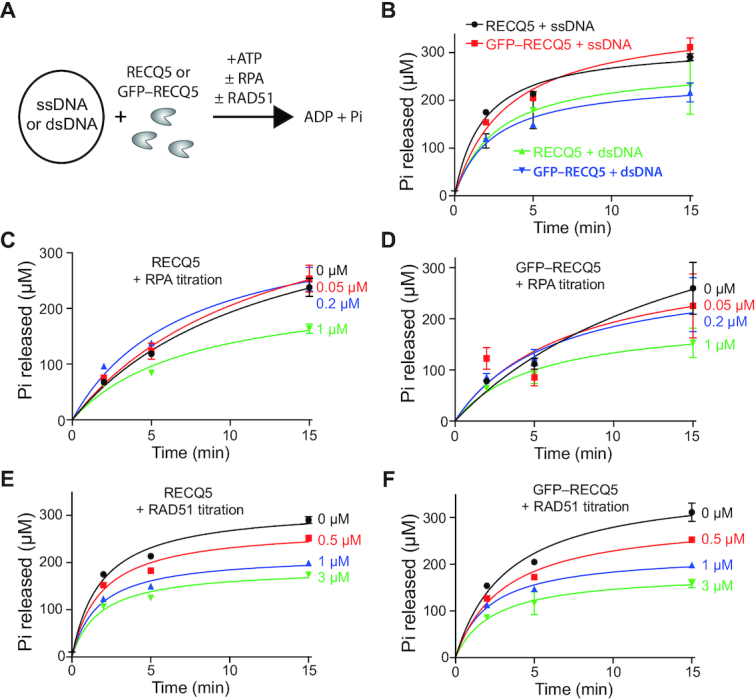
Biochemical characteristics of RECQ5 and GFP–RECQ5. (**A**) Schematic for ATP hydrolysis assays comparing RECQ5 and GFP–RECQ5 (10 nM) in reactions with either ssDNA or dsDNA and with or without RPA or RAD51 (in assays with ssDNA only). (**B**) RECQ5 (10 nM) and GFP–RECQ5 (10 nM) in reactions with either M13 ssDNA (2 μM nucleotides) or pUC19 dsDNA (2 μM nucleotides). (**C**) RECQ5 (10 nM) ATPase assays in the presence of 0, 0.05, 0.2 and 1.0 μM RPA, as indicated. (**D**) GFP–RECQ5 (10 nM) ATPase assays in the presence of 0, 0.05, 0.2 and 1.0 μM RPA, as indicated. (**E**) RECQ5 (10 nM) ATPase assays in the presence of 0, 0.5, 1.0 and 3.0 μM RAD51, as indicated. Also shown is a 3.0 μM RAD51 only control. (**F**) GFP–RECQ5 (10 nM) ATPase assays in the presence of 0, 0.5, 1.0 and 3.0 μM RAD51, as indicated. Error bars represent the standard deviation from three independent experiments.

RECQ5 was expressed and purified as a N-terminally GFP-tagged fusion for use in single molecule assays (see Materials and Methods). This fusion protein retains function *in vivo* (29,30) and biochemical assays confirmed that purified GFP–RECQ5 retained ATP hydrolysis activity comparable to untagged RECQ5 on naked ssDNA, naked dsDNA, RPA–ssDNA and RAD51–ssDNA (Figure 1A–F, Supplementary Table S1 and Supplementary Figure S1); note that the ATPase activity of RAD51 is negligible under these reaction conditions (Figure 1E). We conclude that GFP-tagged RECQ5 retains biochemical properties that are similar to untagged RECQ5. These ATPase assays, and in all of the ssDNA curtain assays used below, were conducted in the presence of 1 mM MgCl_2_ and 5 mM CaCl_2_. The presence of calcium is necessary for optimal activity of the human RAD51 filaments under our *in vitro* reaction conditions (46) and in the absence of Ca^2+^ RAD51 spontaneously dissociates from ssDNA (47). Although the reason RAD51 requires Ca^2+^*in vitro* remains unknown, it is possible that Ca^2+^ functionally replaces other cellular factors, such as BRCA2 or the RAD51 paralogs, allowing for enhanced RAD51 filament stability in their absence.

**Figure 2.**
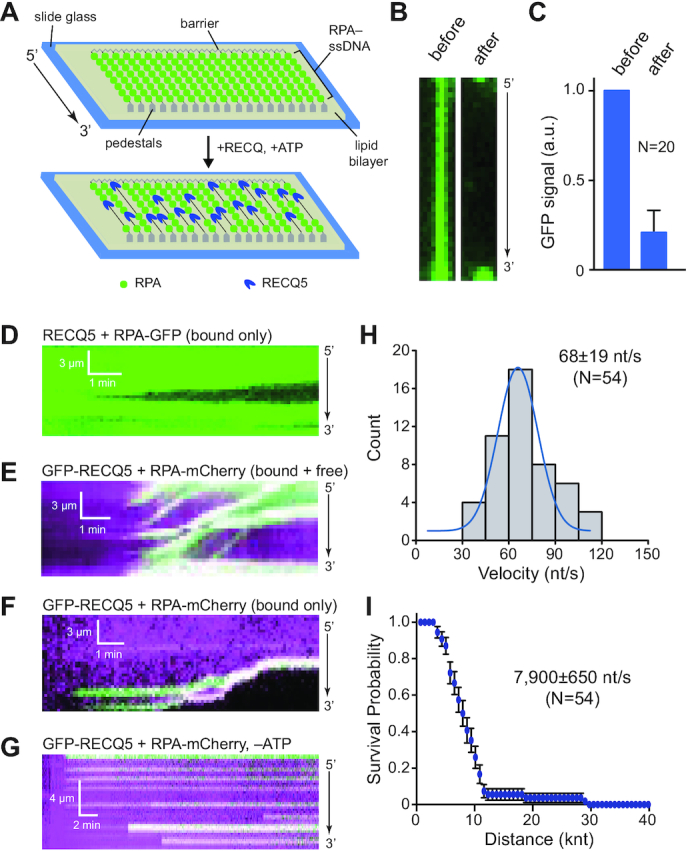
RECQ5 undergoes rapid unidirectional translocation on RPA-coated ssDNA molecules. (**A**) Schematic for visualizing the activity of unlabeled RECQ5 on ssDNA curtains bound by RPA–GFP; in these assays, the movement of RECQ5 (unlabeled) is revealed by the loss of RPA–GFP (green) from the ssDNA because there is no free RPA–GFP in solution. (**B**) Wide-field images showing examples of RPA–GFP (green) bound ssDNA molecules before and after the addition of unlabeled RECQ5. (**C**) Quantitation of normalized RPA–GFP signal (integrated over the entire length of the ssDNA substrates) before and after addition of unlabeled RECQ5. Error bars represent the 68% confidence intervals for 20 ssDNA molecules. (**D**) Kymograph showing the progressive 3′ to 5′ loss of RPA–GFP signal (green) from a single ssDNA molecule in the presence of 25 nM unlabeled RECQ5. (**E**) Kymograph showing the behavior of GFP–RECQ5 (green) on RPA–mCherry-bound ssDNA (magenta) in the presence of 0.5 nM free RPA–mCherry and 2 mM ATP. (**F**) Kymograph showing the behavior of GFP–RECQ5 (green) on RPA–mCherry-bound ssDNA (magenta) in the absence of free RPA–mCherry but with 2 mM ATP. (**G**) Kymograph showing the behavior of GFP–RECQ5 (green) in the presence of 0.5 nM free RPA–mCherry in buffer lacking ATP. (**H**) Velocity distribution for GFP–RECQ5 on RPA–ssDNA; the blue line is a Gaussian fit to the data. (**I**) Survival probability plot indicating the processivity of GFP–RECQ5 during translocation on RPA–ssDNA. Error bars represent 95% confidence intervals calculated from bootstrap analysis, and the reported processivity values correspond to the distance at which 50% of the RECQ molecules had stopped or dissociated from the ssDNA.

To visualize unlabeled RECQ5 interactions with RPA-bound ssDNA, we prepared ssDNA curtains bound by RPA–GFP, unbound RPA–GFP was then flushed out of the sample chambers followed by the addition of unlabeled 25 nM RECQ5 (Figure 2A); note, that unless otherwise indicated, all reactions with RECQ5 contained 2 mM ATP. When incubated with the RPA–ssDNA molecules, RECQ5 cleared the RPA–GFP from the ssDNA (Figure 2B and C). Inspection of the corresponding kymographs revealed RECQ5 translocation along the ssDNA as evidenced by the progressive loss of RPA–GFP fluorescence signal in the 3′ to 5′ direction (Figure 2D), corresponding to the reported polarity (17). Similar results were observed in reactions using GFP–RECQ5 and RPA–mCherry. Using ssDNA curtains with 25 nM GFP–RECQ5, 0.5 nM RPA–mCherry and 2 mM ATP, we could readily observe GFP–RECQ5 bound to the RPA–mCherry–ssDNA. Bound molecules of GFP–RECQ5 underwent unidirectional 3′ to 5′ movement along the RPA-bound ssDNA substrates (Figure 2E). In addition, reactions performed in the absence of free RPA–mCherry confirmed that GFP–RECQ5 stripped the bound RPA from the ssDNA (Figure 2F). Importantly, assays conducted in the absence of ATP revealed that the ability of RECQ5 to bind tightly to RPA–ssDNA was independent of ATP hydrolysis (Figure 2G). However, the bound RECQ5 complexes exhibited no translocation activity when ATP was omitted from the reactions, and instead remained stationary during the duration of the experimental observations (Figure 2G). This result confirms that the observed movement of RECQ5 on RPA–ssDNA is consistent with expectations for an ATP-dependent ssDNA motor protein. Analysis of the data sets collected for GFP–RECQ5 in the presence of 2 mM ATP and 0.5 nM free RPA–mCherry (Figure 2E) revealed a translocation velocity of 68 ± 19 nt/s (N = 54) and an average processivity of 7900 ± 650 nt (*N* = 54; Figure 2H–I & Supplementary Figure S2). Taken together, our results show that RECQ5 is a robust ssDNA translocase that can readily interact with RPA-bound ssDNA, suggesting that RECQ5 might interact with similar substrates while fulfilling its genome maintenance functions during DNA replication and repair.

### BLI assays show that RECQ5 efficiently disrupts RAD51–ssDNA filaments

**Figure 3.**
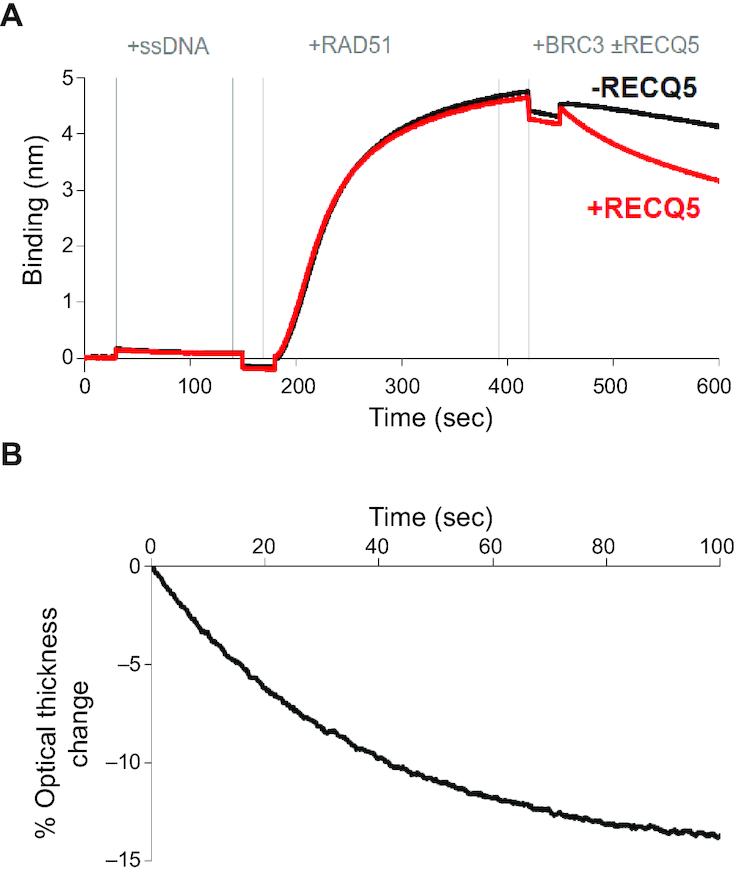
BLI assay for RECQ5 removal of RAD51 from ssDNA. (**A**) Sensorgram of biolayer interferometry assay consisting of five steps: (i) initial wash with BLI buffer starting at 0 s, (ii) loading of 5′ biotinylated dT43-mer (40 nM) at 30 s, (iii) wash with BLI buffer at 150 s, (iv) association step with 2 μM RAD51 protein at 180 s to form presynaptic filament, (v) wash with BLI buffer at 420 s and (vi) dissociation step with 2 μM BRC3 without (black line) or with (red line) 40 nM RECQ5 at 450 s. (**B**) The data points from dissociation phase shown in (A) were normalized to the starting point, the data for BRC3 alone were then subtracted from data including RECQ5. The % optical signal change is plotted as a function of time. This plot shows the difference between the dissociation phase for the plus and minus RECQ5 data sets from panel (A). For this, the data points from dissociation phase in (A) were normalized to the starting point, then the data for the BRC3 alone reaction (minus RECQ5) were subtracted from the data from the reaction that included RECQ5. The % optical signal change is plotted as a function of time.

Previous studies have shown that RECQ5 can act as an antirecombinase by removing RAD51 from ssDNA *in vitro* (21,25), and this activity has been implicated in the SDSA pathway of HR (21) as well as in RECQ5-mediated stabilization of stalled replication forks (30). However, the complete mechanistic understanding of the RECQ5 antirecombinase activity has not yet been provided. We established a biolayer interferometry (BLI) assay to examine interactions between RECQ5 and the RAD51–ssDNA filament (48). BLI method monitors the interference pattern of white light, which is affected by the thickness of the immobilized biomolecule layer. We used streptavidin biosensor tips to immobilize a biotinylated ssDNA substrate which was then bound by RAD51 (see Materials and Methods) allowing us to then test for RAD51 removal by RECQ5. To prevent RAD51 protein from rebinding to the ssDNA after its removal, we used a previously described system of outcompeting this re-binding by sequestering dissociated RAD51 monomers in free solution through addition of a conserved BRC repeat region (BRC3) of BRCA2 protein (49); note that all BLI experiments looking at RECQ5-mediated removal of RAD51 from ssDNA include the BRC3 peptide. The data from the time point where the BRC3 peptide was added to the RAD51–ssDNA nucleoprotein filaments in the absence or presence of RECQ5 (Figure 3A), were normalized to starting point and the amplitude of the change was plotted as a function of time (Figure 3B). From the curve of this dissociation step, we can observe a fast initial decreasing phase of RAD51 removal from DNA, which is dependent on RECQ5, suggesting rapid RAD51 dissociation from ssDNA (Figure 3A), whereas the second phase represents slow destabilization corresponding to BRC3-dependent and RECQ5-independent RAD51 dissociation (Figure 3A).

### Single molecule visualization of RECQ5 antirecombinase activity

**Figure 4.**
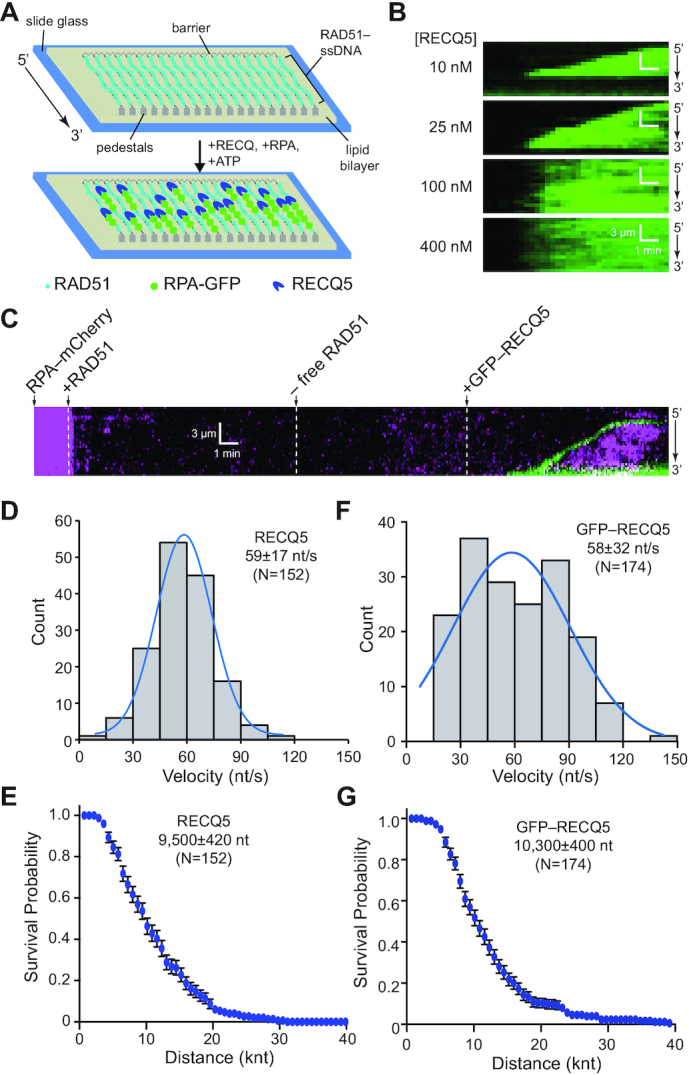
DNA curtain assays showing RECQ5 strips RAD51 from ssDNA. (**A**) Schematic illustration depicting the RECQ5-mediated removal of unlabeled RAD51 from ssDNA allowing its replacement by a fluorescently-tagged variant of RPA. (**B**) Kymographs showing the disruption of the RAD51 filament after addition of 10, 25, 100 or 400 nM RECQ5 (unlabeled) with RPA–GFP. (**C**) Kymograph showing the assembly of a RAD51 filament (unlabeled) on an ssDNA molecule coated with RPA–mCherry (magenta), followed by disruption of the RAD51 filament after addition of GFP–RECQ5 (green). (**D**) Velocity distribution for unlabeled RECQ5 during disruption of RAD51 filaments; the blue line is a Gaussian fit to the data. (**E**) Survival probability plot indicating the processivity of unlabeled RECQ5 during RAD51 filament disruption. Error bars represent 95% confidence intervals calculated from bootstrap analysis. (**F**) Velocity distribution for GFP–RECQ5 during disruption of RAD51 filaments; the blue line is a Gaussian fit to the data. (**G**) Survival probability plot indicating the processivity of GFP–RECQ5 during RAD51 filament disruption. Error bars represent 95% confidence intervals calculated from bootstrap analysis.

To directly examine RECQ5 antirecombinase activity at the single molecule level, we prepared RAD51–ssDNA curtains using unlabeled RAD51, as described previously (Figure 4A) (35,39,50). Of note, we have previously shown that the resulting RAD51 filaments are extremely stable and do not spontaneously dissociate unless ATP and Ca^2+^ are flushed from the sample chamber (47). We then conducted assays with either unlabeled RECQ5 and RPA–GFP or GFP–RECQ5 and RPA–mCherry. Our expectation for these assays was that the removal of RAD51 by RECQ5 should result in the appearance of fluorescently-tagged RPA on the ssDNA and the fluorescent RPA should spread in the 3′ to 5′ direction commensurate with the translocation characteristics of RECQ5 (Figure 4A). Consistent with these expectations, inspection of the resulting data sets for assays with unlabeled RECQ5 (10–400 nM) and 0.5 nM free RPA–GFP revealed that the RAD51 dissociated from the ssDNA and was quickly replaced by RPA–GFP (Figure 4B). At 100 and 400 nM RECQ5 the RPA–GFP began appearing at multiple locations along the ssDNA, consistent with the interpretation that multiple molecules of RECQ5 bound at spatially distinct sites along the ssDNA molecule and began stripping RAD51 from the ssDNA (Figure 4B). In contrast, at lower concentrations of RECQ5 (10 and 25 nM) we could readily observe individual events in which RPA–GFP initially appeared at single sites on the ssDNA then appeared to spread in the 3′ to 5′ direction along the ssDNA, consistent with the expectation that these cases represented the action of single RECQ5 complexes (Figure 4B).

Assays conducted with 25 nM GFP–RECQ5 and RPA–mCherry demonstrated that RECQ5 was indeed positioned at the 5′ end of the expanding tracts of RPA, confirming that the appearance of RPA coincided with the movement of RECQ5 along the RAD51-bound ssDNA molecules (Figure 4C). Analysis of data sets conducted with unlabeled RECQ5 revealed an average velocity of 59 ± 17 nt/s (*N* = 152; Figure 4D) and processivity of ∼9500 ± 420 nt before stopping or dissociating from the ssDNA (*N* = 152; Figure 4E & Supplementary Figure S2). Similar to unlabeled RECQ5, the GFP–RECQ5 translocated at a velocity of 58 ± 32 nt/s (*N* = 174; Figure 4F) with an average processivity of 10 300 ± 400 nt (*N* = 174; Figure 4G & Supplementary Figure S2). Assuming a ratio of 1 RAD51 monomer per three nucleotides (9,51), these values correspond to the removal of ∼20 RAD51 monomers per second and total of ∼3300 RAD51 monomers per translocation event. Taken together, these results confirm that RECQ5 readily dismantles ATP-bound RAD51 filaments.

### RECQ5 forms multimeric complexes during translocation

To examine the oligomeric state of the active unit of GFP–RECQ5 as it was undergoing translocation on RAD51–ssDNA filaments we performed photobleaching analysis. In these assays, GFP–RECQ5 translocation was initiated on RAD51–ssDNA, as described above, using imaging conditions intentionally designed to minimize photobleaching by limiting laser exposure to the sample. Specifically, a single 100-ms frame was collected at 10 s intervals and the laser was shuttered between each frame (see Materials and Methods section; Supplementary Figure S3A). Once actively translocating GFP–RECQ5 molecules were bound to the RAD51–ssDNA, data collection was continued without shuttering the laser, causing the GFP–RECQ5 molecules to photobleach (Supplementary Figure S3A). The GFP signal intensity was then analyzed for photobleaching steps, with each step reflecting the presence of one GFP–RECQ5 molecule (Supplementary Figure S3B). Interestingly, some of the observed GFP–RECQ5 complexes displayed single photobleaching steps (13.6%, *N* = 3/22), however, most of the complexes exhibited two or more photobleaching steps (86.4%, *N* = 19/22), consistent with the conclusion that RECQ5 can form higher order multimers while translocating on ssDNA (Supplementary Figure S3C). Interestingly, AFM volumetric analysis suggested that the RECQ5 proteins were monodisperse in solution and also while bound to a short 90-nt ssDNA fragment (Supplementary Figure S3D–G). These findings suggest the possibility that RECQ5 only oligomerizes upon association with the longer protein-bound ssDNA substrates used in the ssDNA curtain assays, or RECQ5 many not form specific oligomers on the longer ssDNA but may instead preferentially form short tandem arrays (i.e. two of more molecules of RECQ5 bound near one another on the ssDNA, but not interacting through specific protein–protein contacts), similar to what we have previously reported for the Srs2 helicase (42).

### RECQ5 interaction with RAD51 promotes presynaptic filament disruption

**Figure 5.**
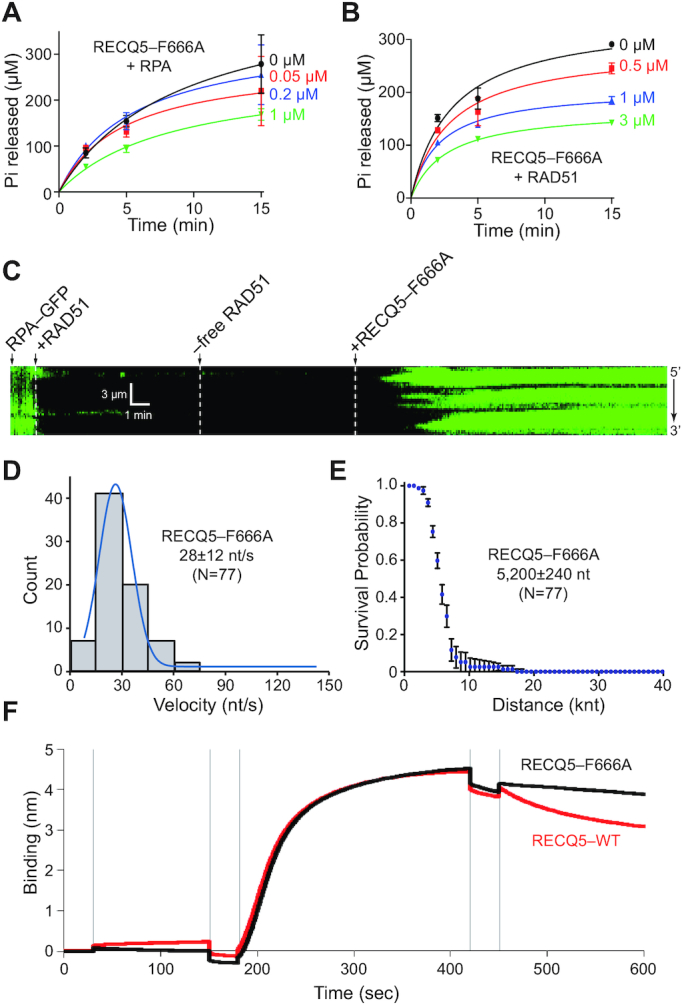
RECQ5–F666A is defective for RAD51 filament disruption. (**A**) RECQ5–F666A (10 nM) ATPase assays in the presence of 0, 0.05, 0.2 and 1.0 μM RPA, as indicated. Error bars represent standard deviation from three independent experiments. (**B**) RECQ5–F666A (10 nM) ATPase assays in the presence of 0, 0.5, 1.0 and 3.0 μM RAD51, as indicated. Error bars represent the standard deviation from three independent experiments. (**C**) Kymograph showing the action of unlabeled RECQ5–F666A (25 nM) on RAD51–ssDNA (unlabeled) in the presence of 0.5 nM free RPA–GFP (green). (**D**) Velocity distribution for RECQ5–F666A on RAD51–ssDNA; the blue line is a Gaussian fit to the data. (**E**) Survival probability plot indicating the processivity of unlabeled RECQ5–F666A during RAD51 filament disruption. Error bars represent 95% confidence intervals calculated from bootstrap analysis. (**F**) Sensogram of BLI assay consisting of five steps: (i) initial wash with BLI buffer at 0 s, (ii) loading of 5′ biotinylated dT43-mer (40 nM) at 30 s, (iii) wash with BLI buffer at 150 s, (iv) association step with 2 μM RAD51 protein at 180 s to form presynaptic filament, (v) wash with BLI buffer at 420 s and (vi) dissociation step with 2 μM BRC3 and 40 nM RECQ5-WT (red) or RECQ5–F666A (black) at 450 s.

RECQ5 physically interacts with RAD51 and this interaction is necessary for RECQ5 to efficiently remove RAD51 from ssDNA (24,25). Mutation of the RECQ5 amino phenylalanine 666 to alanine (F666A) abrogates RECQ5 interactions with RAD51 and prevents RECQ5 from efficiently removing RAD51 from ssDNA (24,25). Therefore, we next examined the influence of the RECQ5–F666A mutant on RAD51 filament disruption in our ssDNA curtain assays. Unlabeled RECQ5–F666A retained ATP hydrolysis activity in the presence of either RPA or RAD51 (Figure 5A–B) and also promoted disruption of RAD51 filaments (Figure 5C), however, this mutant protein displayed a translocation velocity of just 28 ± 12 nt/s (*N* = 77; Figure 5D) and a processivity of 5200 ± 240 nt (*N* = 77; Figure 5E). Highlighting the importance of the RECQ5–RAD51 interactions, these values for RECQ5–F666A are the lowest velocity and processivity that we observed for RECQ5 under any condition tested (Supplementary Figure S2). Similarly, the RECQ5–F666A mutant was not able to destabilize the RAD51 nucleoprotein filaments in BLI assays (Figure 5F), further highlighting the importance of the RAD51–RECQ5 protein interaction in regulating RECQ5-mediated disruption of RAD51 filaments. We note that there is a qualitative difference between the ssDNA curtain assay where RECQ5–F666A strips RAD51 from ssDNA, albeit ∼50% more slowly than wtRECQ5, and the BLI assays which suggest that the F666A mutation might have a greater effect on RAD51 removal (cf. Figure 5C–D and Figure 5F). The discrepancy could result from different DNA substrates. The ssDNA curtains use substrates that are tens of kilobases in length whereas the ssDNA in the BLI assays is just 43 nucleotides in length. The longer DNA may allow for more efficient RECQ5 binding, thus enhancing RAD51 removal.

### ATP hydrolysis by RAD51 is not necessary for RECQ5-mediated removal

The DNA-binding activity of RAD51 and other RecA family members is coupled with ATP binding and hydrolysis. RAD51 filaments assemble onto DNA in the ATP-bound state, and dissociate from DNA after the ATP is hydrolyzed to ADP plus Pi (8,9). The yeast antirecombinase Srs2 takes advantage of this ATP binding/hydrolysis cycle and promotes Rad51 removal from ssDNA by stimulating the ATPase activity of yeast Rad51 (42,52). As a consequence, the yeast Rad51–K191R mutant, which binds but does not hydrolyze ATP (53,54), is more difficult for Srs2 to remove from ssDNA (42,52). The human RAD51–K133R mutant can bind ATP, but is defective for ATP hydrolysis and therefore binds tightly to ssDNA (38), mimicking the yeast K191R mutant. In contrast to Srs2, previous bulk biochemical studies have shown that RECQ5 can prevent D-loop formation in reactions with RAD51–K133R and can remove RAD51–K133R from ssDNA, suggesting that RECQ5 does not couple RAD51 filament disruption to the RAD51 ATP binding/hydrolysis cycle (21).

We conducted BLI assays to extend these previous observations and quantitatively analyze the role of RAD51-mediated ATP hydrolysis during disruption by RECQ5. The data from the time period where the BRC3 peptide was added to the wtRAD51 or RAD51–K133R nucleoprotein filaments in the presence or absence of RECQ5 were normalized to the starting point and the amplitude of the change was plotted as a function of time. The data suggest that RECQ5 can rapidly disrupt the RAD51–K133R nucleoprotein filaments (Supplementary Figure S4A), suggesting that it does not need to stimulate ATP hydrolysis within the RAD51 nucleoprotein filament to promote its disassembly. Interestingly, RECQ5 can clear the RAD51–K133R nucleoprotein filaments even more efficiently than wtRAD51 filaments in these BLI measurements. This effect is likely caused by the fact that RAD51–K133R nucleoprotein filaments are more prone to destabilization by addition of the BRC3 repeats than wtRAD51 (Supplementary Figure S4B), indicating a possible role of monomer–monomer interactions in filament disassembly. Note that all of these assays used the BRC3 peptide to prevent reassociation of RAD51 with the ssDNA after disruption by RECQ5 (see Materials and Methods) and control experiments confirmed that the BRC3 peptide bound similarly to RAD51–K133R, as well as the other RAD51 variants discussed below (Supplementary Figure S4E).

**Figure 6.**
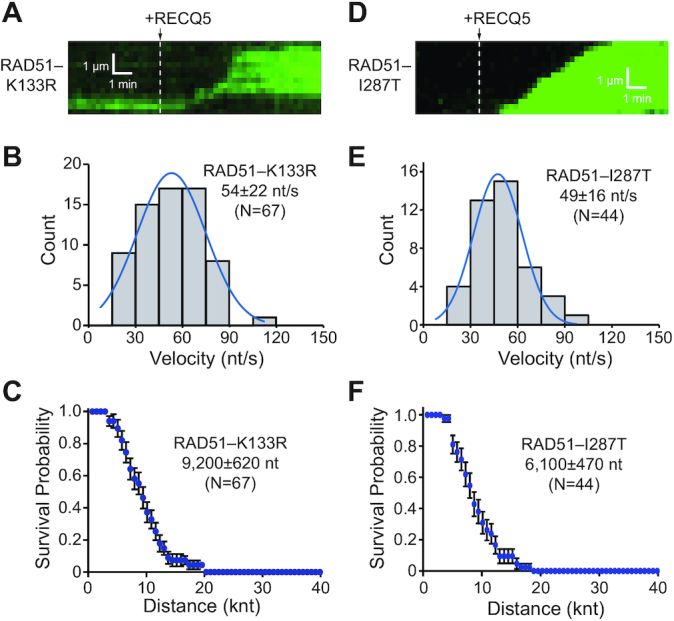
RECQ5 can remove RAD51–K133R and RAD51–I287T from ssDNA. (**A**) Kymograph showing unlabeled RECQ5 (25 nM) acting on ssDNA-bound RAD51–K133R filaments in the presence of free 0.5 nM RPA–GFP (green). (**B**) Velocity distribution for unlabeled RECQ5 on RAD51–K133R–ssDNA; the blue line is a Gaussian fit to the data. (**C**) Survival probability plot indicating the processivity of unlabeled RECQ5 during RAD51–K133R filament disruption. Error bars represent 95% confidence intervals calculated from bootstrap analysis. (**D**) Kymograph showing unlabeled RECQ5 (25 nM) acting on ssDNA-bound by RAD51–I287T filaments in the presence of free 0.5 nM RPA–GFP (green). (**E**) Velocity distribution for unlabeled RECQ5 on ssDNA bound by RAD51–I287T filaments; the blue line is a Gaussian fit to the data. (**F**) Survival probability plot indicating the processivity of unlabeled RECQ5 during RAD51–I287T filament disruption. Error bars represent 95% confidence intervals calculated from bootstrap analysis.

RECQ5-mediated disruption of RAD51–K133R filaments was also directly assessed in ssDNA curtain assays. Consistent with data from the BLI assays, RECQ5 could readily remove RAD51–K133R from ssDNA (Figure 6A), yielding a translocation velocity of 54 ± 22 nt/s (*N* = 67; Figure 6B) and processivity of 9500 ± 620 nt (*N* = 67; Figure 6C), which were comparable to the translocation characteristics of RECQ5 in reactions with wtRAD51 (Supplementary Figure S2). These findings are consistent with previous studies (21,25) and confirm that RECQ5 can readily remove RAD51–K133R from ssDNA.

To determine whether RECQ5 preferentially removes the nucleotide-bound form RAD51 from ssDNA, we decided to also test the Walker box RAD51–K133A mutant, which is defective for ATP hydrolysis and is thought to be defective in ATP-binding, although this latter point has not been rigorously validated (38). RAD51–K133A is incapable of strand exchange activities *in vitro* (38), but can bind ssDNA and dsDNA in solution (38) and the resulting nucleoprotein filaments are highly dynamic (36), therefore cannot be monitored in the D-loop assays. We used the BLI assays to examine the effect of RECQ5 on nucleoprotein filaments made with RAD51–K133A in the absence of RPA. Interestingly, the observed change in optical thickness after addition of RECQ5 was comparable for wtRAD51 and RAD51–K133A nucleoprotein filaments (Supplementary Figure S4C), implying that RECQ5 can disrupt also nucleotide-free RAD51–DNA complexes. These findings confirm that RECQ5 can readily remove nucleotide-bound or nucleotide-free RAD51 from ssDNA.

### Human RAD51–I287T is readily removed from ssDNA by RECQ5

Rad55–Rad57 paralog complex is required to modulate yeast Rad51 protein activity and is thought to counteract the antirecombinase activity of Srs2. The yeast Rad51 gain-of-function mutant Rad51–I345T was isolated as a suppressor mutation that partially bypassed the requirement for Rad55–Rad57 paralog complex (55), suggesting that the Rad51–I345T mutant might be more resistant to Srs2 (55). Consistent with this hypothesis, single molecule studies revealed that Rad51–I345T assembled more quickly onto ssDNA and yielded more stable filaments that dissociate more slowly, which were also more resistant to the effects of Srs2 (42). We sought to determine whether a comparable gain-of-function mutant with enhanced DNA-binding characteristics could be made for human RAD51. Based on sequence homology, we generated the mutant protein RAD51–I287T, which is known to bind to DNA better than wild-type RAD51 (56). Surprisingly, RECQ5 could easily remove RAD51–I287T from ssDNA (Figure 6D), yielding a translocation velocity of 49 ± 16 nt/s (*N* = 44; Figure 6E) and a processivity of 6100 ± 470 nt (*N* = 67; Figure 6F); these values were reduced compared to reactions with wtRAD51 (Supplementary Figure S2). To confirm these observations, we also analyzed RECQ5-mediated disruption of the RAD51–I287T nucleoprotein filament using BLI measurements. Indeed, these experiments demonstrated that RECQ5 removes RAD51–I287T from ssDNA less efficiently compared to wtRAD51 (Supplementary Figure S4D). Taken together, we conclude that although human RAD51–I287T forms more stable filaments than wtRAD51, the resulting filaments remain susceptible to disruption by RECQ5.

### RECQ5-mediated disruption of DMC1 filaments

**Figure 7.**
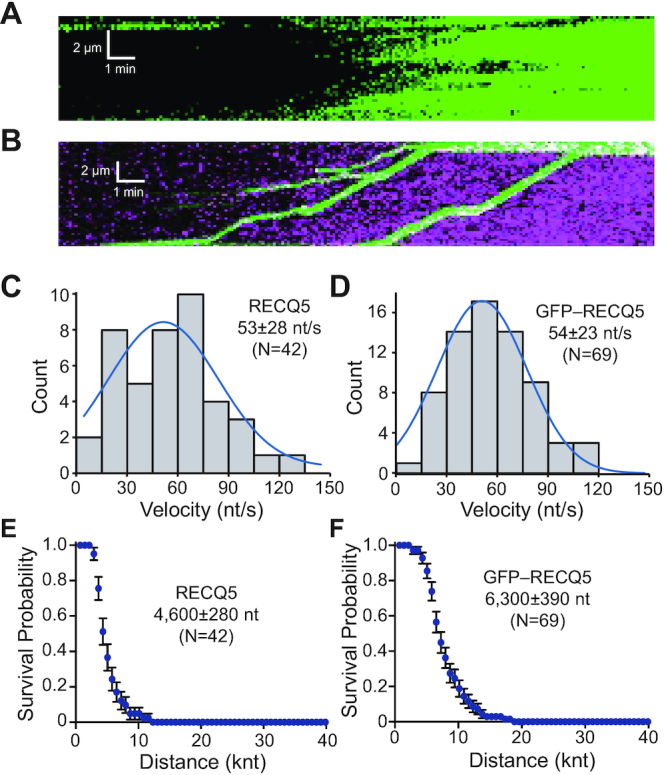
DMC1 reduces the processivity of RECQ5. (**A**) Kymograph showing unlabeled RECQ5 (25 nM) acting on DMC1–ssDNA filaments in the presence of free 0.5 nM RPA–GFP (green). (**B**) Kymograph showing GFP–RECQ5 (25 nM; green) acting on DMC1–ssDNA filaments in the presence of free 0.5 nM RPA–mCherry (magenta). (**C**) Velocity distribution for unlabeled RECQ5 on DMC1–ssDNA; the blue line is a Gaussian fit to the data. (**D**) Velocity distribution for GFP–RECQ5 on DMC1–ssDNA; the blue line is a Gaussian fit to the data. (**E**) Survival probability plot indicating the processivity of unlabeled RECQ5 during DMC1 filament disruption. Error bars represent 95% confidence intervals calculated from bootstrap analysis. (**F**) Survival probability plot indicating the processivity of GFP–RECQ5 during DMC1 filament disruption. Error bars represent 95% confidence intervals calculated from bootstrap analysis.

Most eukaryotes have two distinct members of RecA family recombinases, Rad51 which is expressed in mitosis and meiosis, and the meiosis-specific Dmc1 protein (57,58). Interestingly, yeast Dmc1 blocks the antirecombinase activities of Srs2 and Sgs1, suggesting that meiotic recombination intermediates containing Dmc1 may not be dismantled by these enzymes (41,59,60). Therefore, we sought to determine whether RECQ5 could remove human DMC1 from ssDNA. Control assays confirmed that human DMC1 could form stable filaments in our ssDNA curtain assays, but under similar reaction conditions assembles ∼82% more slowly (*k*_on,DMC1_ = 1.7 ± 0.3 × 10^–3^ s^–1^) and dissociates ∼76% more slowly (*k*_off,DMC1_ = 2.7 ± 0.57 × 10^–3^ s^–1^) compared to RAD51 (Supplementary Figure S5). In contrast to yeast Srs2 and Sgs1 (41,59,60), RECQ5 could readily dismantle DMC1 filaments (Figure 7A–B). The velocity of unlabeled RECQ5 (53 ± 28 nt/s, *N* = 41; Figure 7C) and GFP–RECQ5 (54 ± 23 nt/s, *N* = 69; Figure 7D) were largely unaffected in reactions with DMC1 compared to reactions with RAD51, despite the fact that DMC1 alone dissociates more slowly from ssDNA than RAD51 (Supplementary Figure S2). Interestingly, the processivities of unlabeled RECQ5 and GFP–RECQ5 on DMC1–ssDNA filaments were both significantly reduced compared to reactions with RAD51 (Supplementary Figure S2), yielding values of 4600 ± 280 nt (*N* = 42) and 6300 ± 390 nt (*N* = 69) for RECQ5 and GFP–RECQ5, respectively (Figure 7E and F). These reductions in processivity in reactions with DMC1 were comparable to reactions performed with RAD51 using the RAD51 interaction deficient mutant RECQ5–F666A (Supplementary Figure S2). These findings indicate that RECQ5 may not interact specifically with DMC1 and is consequently less optimal at disrupting DMC1 filaments compared to RAD51 filaments.

**Figure 8.**
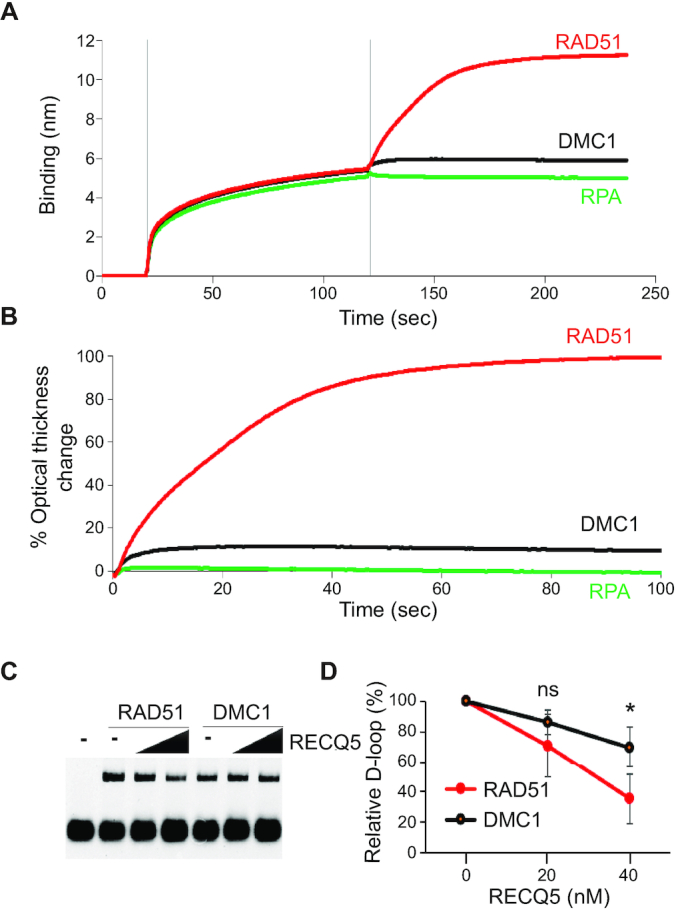
RECQ5 interaction affinities with RAD51, DMC1 and RPA. (**A**) Sensogram of BLI assay consisting of three steps: (i) initial wash with BLI buffer at 0 s, (ii) loading of 0.5 μg/ml rabbit polyclonal RECQ5 antibody pre-incubated with 1.5 μM RECQ5 at 20 s, (iii) association step with 2.5 μM RAD51 (red), DMC1 (black) or RPA (green) protein at 120 s. The binding affinity of the proteins was measured as a increase of the optical thickness of the biomolecule layer in nanometers (nm). (**B**) Traces of optical thickness change after addition of indicated proteins. The data from the time period where indicated proteins were added to the RECQ5 protein-bound to RECQ5 antibody, were normalized to the RAD51 protein trace. (**C**) D-loop disruption assay. RAD51 (2 μM) or DMC1 (2 μM) was bound to 40 nM 90-mer ssDNA for 10 min at 37°C followed by the addition of RECQ5 (20 and 40 nM) and RPA (300 nM) for 5 min at 37°C. Then, HOP2–MND1 (300 nM) was added to the mixture for 2 min, followed by addition of supercoiled plasmid DNA containing homologous region to the oligonucleotide for 10 min at 37°C. **(D)** Quantification of D-loop assay. The percentage of RECQ5-mediated D-loop was normalized to the sample without RECQ5 and graphed as the average of triplicates ± SD (*P< 0.05).

To help explain observed differences in processivity of RECQ5 on DMC1 and RAD51 nucleoprotein filaments, we examined their interaction affinities using BLI measurements (Figure 8A–B and Supplementary Figure S6). Here, we immobilized RECQ5 to a biosensor coated with protein A using an anti-RECQ5 antibody. We then added either RAD51, DMC1 or RPA and measured the relative binding association for each protein to RECQ5 as an increase in thickness of the biomolecule layer (Figure 8A and B). After adding RAD51 protein (2.5 μM) to the immobilized RECQ5 (1.5 μM) we detected an increase in thickness by 5.7 nm, confirming the previous report of a direct protein–protein interaction between RECQ5 and RAD51 (21,25). In striking contrast to RAD51, after addition of DMC1, we observed increase in thickness of just 0.4 nm, indicating only weak binding of DMC1 to RECQ5. Finally, addition of RPA to the immobilized RECQ5 did not result in any change in optical thickness compared to control with buffer, suggesting no direct protein interaction between these two proteins (Figure 8A and B).

Next, we wanted to address whether the RECQ5 could affect DMC1 nucleoprotein filament during D-loop formation (Figure 8C and D). We incubated fluorescently labeled DNA with DMC1 protein to form a DMC1 nucleoprotein filament, followed by incubation with RECQ5 and RPA. Then Hop2-Mnd1 was added to the nucleoprotein filament and reaction was started by adding pBluescript containing complementary sequence to the fluorescent ssDNA. The data shows that RECQ5 can inhibit the D-loop formation in a concentration dependent manner on both DMC1 and RAD51 nucleoprotein filaments. Interestingly, RECQ5 displays a lower inhibitory effect on DMC1 in D-loop disruption assay when compared to the RAD51. This result is consistent with our previous data and suggests that DMC1 nucleoprotein filament is less prone to clearance by RECQ5 than RAD51 and may reflect a weaker binding between DMC1 and RECQ5.

### RECQ5 does not translocate on dsDNA

**Figure 9.**
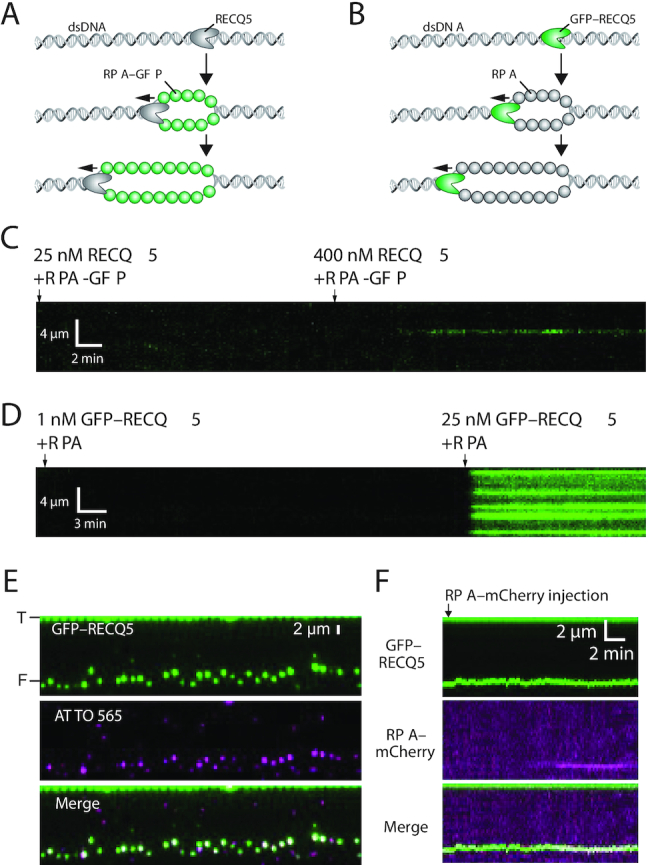
RECQ5 does not processively unwind dsDNA intermediates. (**A**) Schematic illustration showing assay for detecting dsDNA unwinding by unlabeled RECQ5 in the presence of RPA–GFP. (**B**) Schematic illustration showing assay for detecting dsDNA unwinding by GFP–RECQ5 in the presence of unlabeled RPA. (**C**) Kymograph showing that unlabeled RECQ5 does not produce detectable ssDNA products when incubated with a dsDNA substrate (lambda-DNA; 48 502 kbp) anchored both ends to the flow cell surface. The location of the dsDNA is first verified by labeling with YOYO-1 (not shown), the YOYO-1 was then removed by a brief injection of 5 mM NaCl. The dsDNA was then incubated with sequential injections of 25 and 400 nM unlabeled RECQ5 in the presence of 1 nM free RPA–GFP (green), as indicated. (**D**) Kymograph showing GFP–RECQ5 reactions with dsDNA (lambda-DNA; 48 502 kbp) anchored by both ends to the flow cell surface. The dsDNA incubated with sequential injections of 1 nM and 25 nM GFP–RECQ5 (green) in the presence of 1 nM free RPA (unlabeled), as indicated. (**E**) Assay showing GFP–RECQ5 (green) binding to the free ends of dsDNA substrate (lambda-DNA; 48 502 kbp) anchored by just one end to the flow cell surface. The free end of the DNA has a 30-nt 3′ ssDNA overhang that is labeled with a single ATTO565 fluorophore (magenta), prepared as previously described (64), and ‘T’ and ‘F’ denote the tethered and free ends of the DNA. (**F**) Example kymograph showing that the end-bound RECQ5 shows no evidence for spatial displacement (within optical resolution limits) but some very limited unwinding may be occurring as evidenced by co-localization of RPA–mCherry with the GFP–RECQ5 at the free DNA end.

Our data demonstrate that RECQ5 exhibits robust translocase activity on RPA-, RAD51- and DMC1-bound ssDNA. Using double-stranded DNA curtains, we next sought to determine whether RECQ5 could translocate on lambda-DNA molecules (48 502-bp) that were tethered by both ends to the flow cell surface (35,61). In these assays, we used either unlabeled RECQ5 and RPA–GFP or GFP–RECQ5 and unlabeled RPA. If unlabeled RECQ5 could unwind the dsDNA then we predicted this activity would be revealed by the binding of fluorescent RPA to the resulting ssDNA strands, as we have previously shown for yeast Sgs1 and human BLM helicases (Figure 9A) (35,62). Assays with GFP–RECQ5 and unlabeled RPA cannot directly reveal unwinding activity, but they can be used to determine whether RECQ5 can bind to and translocate on dsDNA (Figure 9B). In striking contrast to results with either RPA–ssDNA, RAD51–ssDNA and DMC1–ssDNA, we found no evidence of efficient RECQ5 translocation as would be evidenced by expanding tracts of ssDNA-bound RPA–GFP (Figure 9A) on naked dsDNA molecules even in assays containing up to 2.5-fold more (25 nM) or 40-fold more unlabeled RECQ5 (400 nM) relative to typical assays with reactions with ssDNA substrates (Figure 9C). In assays with 1 nM GFP–RECQ5 and unlabeled RPA, we were unable to detect efficient binding of GFP–RECQ5 to the dsDNA, indicating that RECQ5 does not bind as well to dsDNA compared to ssDNA substrates (cf. Figures 2E–G and 9D). However, we could detect dsDNA-binding activity when the concentration was increased to 25 nM GFP–RECQ5. It should be noted, that with GFP–RECQ5 we cannot test comparably high concentrations as in reactions with unlabeled RECQ5 (*e.g*. 100 or 400 nM) due to signal background (Figure 9D). Remarkably, the GFP–RECQ5 molecules that were bound to the dsDNA remained completely stationary and yielded no evidence of ATP-dependent translocation activity (Figure 9D). Interestingly, RECQ5 is a DNA-dependent ATPase and its ATP hydrolysis activity is stimulated by both ssDNA and dsDNA (Figure 1B) (63). Thus, although dsDNA can stimulate RECQ5 ATP hydrolysis activity, RECQ5 is not able to unwind or translocate on the dsDNA substrates. Taken together, these results suggest that although RECQ5 is highly active on ssDNA substrates, it may not be able to act upon replication or recombination intermediates comprised of dsDNA.

### RECQ5 does not promote long-range unwinding when bound to DNA ends

Next, we asked whether targeting RECQ5 to a free DNA end would enable it to efficiently unwind the DNA molecules. For these assays, we used lambda-DNA molecules (48 502-bp), as described above, however, only one end of the DNA was anchored to the surface. The other end of the DNA was free in solution and was modified to harbor a 30-nt 3′ ssDNA overhang, as previously described (64). We reasoned that GFP–RECQ5 might bind to the ssDNA overhangs, and if capable of unwinding the DNA, we would be able to detect long-range motion of GFP–RECQ5 along the dsDNA and that any unwound DNA should be detectable by the ssDNA binding activity of RPA–mCherry. Indeed, RECQ5 was readily targeted to the ssDNA overhangs at the ends of the dsDNA substrates (Figure 9E), however, we were unable to detect any translocation of the molecules away from the DNA ends within our optical resolution limits (Figure 9F). Notably, many of the DNA ends (94.2%; *N* = 227 of 241) showed evidence of dim fluorescent RPA–mCherry foci by the end of the 20-minute duration measurements with ∼50% of the RPA–mCherry foci appearing at approximately the 6-min time point but the mCherry signal did not increase substantially in intensity over time (Figure 9F). These findings suggest that the end-bound RECQ5 could promote limited unwinding of the dsDNA, but the processivity of these unwinding reactions was much less than the processivity of RECQ5 moving along RPA-, RAD51- or DMC1-ssDNA substrates.

### Heteroduplex DNA blocks RECQ5 translocation

**Figure 10.**
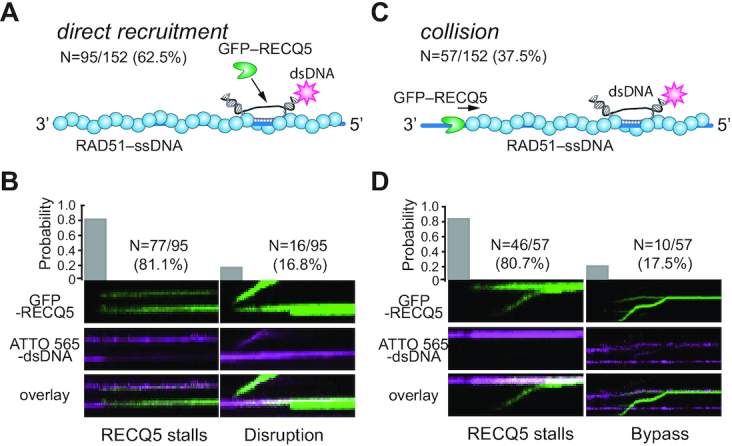
RECQ5 stalls upon encountering heteroduplex DNA joints. (**A**) Schematic illustration showing direct recruitment of RECQ5 to a heteroduplex DNA joint and fraction of total events that occur through direct recruitment. (**B**) Kymographs and probability of occurrence for GFP–RECQ5 (green) stalling after direct recruitment or disrupting the ATTO565-labeled heteroduplex DNA joint (magenta), as indicated. (**C**) Schematic illustration showing RECQ5 colliding with the heteroduplex DNA joint after undergoing 3′ to 5′ translocation and fraction of total events that occur through this pathway. (**D**) Kymographs and probability of occurrence for GFP–RECQ5 (green) stalling or bypassing the heteroduplex DNA joints (magenta), as indicated.

RECQ5 can prevent RAD51-catalyzed DNA-loop formation by disrupting RAD51 filaments before the formation of D-loops, however, RECQ5 is unable to disrupt D-loops after they have already formed (21). To examine this process more closely, we generated heteroduplex DNA joints (D-loops) by incubating the RAD51–ssDNA filaments with an ATTO565-labeled 70-bp dsDNA substrate that contained an internal 15-nt tract of sequence homology to the RAD51-bound ssDNA, as previously described (50,65). We then asked what took place when RECQ5 encountered these heteroduplex DNA joint molecules. These assays revealed two distinct classes of events: (i) RECQ5 could be directly recruited to the heteroduplex DNA joints (62.5% of observed events, *N* = 95/152; Figure 10A and B) or (ii) RECQ5 could bind elsewhere on the RAD51–ssDNA and then translocate in the 3′ to 5′ direction prior to encountering the heteroduplex joint (37.5% of observed events, *N* = 57/152; Figure 10C and D). In both cases, the predominate outcome was that the heteroduplex joint remained intact and blocked any further RECQ5 translocation activity. The majority of events (81.1%, *N* = 77/95) involving direct recruitment of RECQ5 to the heteroduplex joints, RECQ5 bound directly to the heteroduplex joint, but then failed to undergo any observable translocation (Figure 10B). In the remaining direct recruitment events (16.8%, *N* = 16/95) RECQ5 translocated along the RAD51–ssDNA, resulting in disruption of the heteroduplex DNA joint, and the ATTO565-labeled dsDNA fragments appeared to remain bound to RECQ5 as it translocated (Figure 10B). For the majority of cases where RECQ5 translocated prior to encountering the heteroduplex DNA joint (80.7%, *N* = 46/57), the collision with the heteroduplex dsDNA caused RECQ5 to stall and stop translocating (Figure 10D). For the remaining examples (17.5%; *N* = 10/57), RECQ5 bypassed the heteroduplex DNA joint (Figure 10D). The observation that RECQ5 translocation on RAD51–ssDNA is blocked by heteroduplex DNA joints is consistent with previously published biochemical data (21) and is consistent with our observation that RECQ5 is unable to act on naked dsDNA substrates (see above). Together, these findings imply the possibility that the activities of RECQ5 may be confined to ssDNA-replication/recombination intermediates.

## DISCUSSION

Humans have a total of five RecQ helicases, all of which are thought to play important, yet still not well understood, roles in the maintenance of genome integrity (1–7,9). Some of the challenges with more fully defining the functions of these proteins is that they each seem to be involved in multiple processes. The functional consequence of interactions with partner proteins remain poorly characterized, which also seems to have overlapping or partially overlapping roles in genome integrity (1–7,9). Here, we have conducted single molecule and kinetic characterization of the human RecQ helicase RECQ5 and provide new insights into its interactions with substrates mimicking DNA replication and repair intermediates.

### RECQ5 is a robust ssDNA-specific antirecombinase

The yeast helicases Sgs1 and Srs2, and the human helicases RECQ5 and BLM have all been defined as antirecombinases that regulate the outcome of homologous recombination presumably through the ability to disassemble Rad51 filaments and/or the ability to dissolve the recombination intermediates (3,4,6,7). From a mechanistic perspective, although they share many similarities, they also exhibit many differences that may reflect important underlying differences in their biological functions. Thus, it is useful to compare the activities of RECQ5 to other helicases that participate in homologous recombination when considering the specific roles of RECQ5 in DNA metabolism.

Our work shows that human RECQ5 can translocate in the 3′ to 5′ direction on protein-bound ssDNA substrates and travels at a velocity of ∼60–80 nucleotides per second over distances on the order of ∼5–10 kb. In addition, we show that RECQ5 can strip RPA, RAD51 and DMC1 from ssDNA during translocation and its ability to remove RAD51 from ssDNA is not coupled to the RAD51 ATP hydrolysis cycle. The ability of RECQ5 to interact with RAD51 aids in the disruption of RAD51 filaments, as evidenced by the reduced translocation velocity of the RAD51 interaction-deficient mutant RECQ5–F666A. Nevertheless, RECQ5–F666A can still remove RAD51 from ssDNA, indicating that this interaction is beneficial but is not absolutely essential. Our data underline the importance of the mechanism of how RECQ5 acts on RAD51 nucleofilaments. This information may help us to understand the mechanisms of how RECQ5 regulates RAD51 filament assembly during homologous recombination and therein control HR sub-pathway choice (20) and how it removes RAD51 from stalled replication forks at chromosomal fragile sites (CFS) to facilitate their cleavage by MUS81–EME1 (26).

Srs2 is a SF1 (super-family 1) UvrD-like helicase that is considered a prototypical antirecombinase that acts by dismantling Rad51–ssDNA filaments (3,66,67). Srs2 can translocate in the 3′ to 5′ direction ∼140 nts/s over distances of ∼18 kilo-nucleotides (knt) on Rad51-coated ssDNA (42). Srs2 can also translocate on RPA–ssDNA while stripping RPA from the ssDNA, but is unable to act on Dmc1–ssDNA (41,59,68). In contrast to RECQ5, Srs2 takes advantage of the Rad51 ATP hydrolysis to help promote Rad51 filament disruption (52). But like RECQ5, Srs2 is unable to act upon dsDNA substrates, suggesting that its antirecombinase activities seem to be confined to ssDNA containing recombination intermediates.

The yeast mutant Rad51–I345T bypasses the need for the Rad55–Rad57 paralog complex to counteract the antirecombinase activity of Srs2 (55) and Srs2 shows a 40% reduction in translocation velocity on Rad51–I345T filaments compared to wtRad51 filaments (42). Similar to yeast Rad51–I345T (42,69), the corresponding human RAD51–I287T mutant also shows enhanced DNA binding properties (56). While RECQ5 is capable to remove RAD51–I287T from ssDNA, it exhibits a significantly reduced translocation velocity and processivity. Therefore, like its yeast counterpart, the human RAD51–I287T mutant protein can abrogate RAD51 removal from ssDNA. It will be interesting to address in future whether human RAD51–I287T also exhibits a reduced dependence upon the RAD51 paralog complexes.

Sgs1 is one of the two RecQ helicases found in yeast and participates in DNA end resection and Holliday junction dissolution (1,9). Sgs1 can also translocate in the 3′ to 5′ direction ∼30 nts/s over distances of ∼4000 nt on Rad51-coated ssDNA, thus Sgs1 is slower and less processive than RECQ5 (60). Sgs1 can also translocate on RPA–ssDNA, but does not appear to remove RPA from the ssDNA, and like Srs2, Sgs1 is unable to act on Dmc1–ssDNA (60). In striking contrast to both Srs2 and RECQ5, Sgs1 can unwind long (≥2 kb) dsDNA substrates in bulk biochemical assays (70,71) and also exhibits robust activity in single molecule DNA curtain assays with dsDNA substrates (62). Thus, one of the most striking differences between RECQ5 and Sgs1 are their distinct responses to dsDNA substrates. It is likely that the ability of Sgs1 to interact with dsDNA may arise from its participation in DNA end resection and HJ dissolution, both of which would require Sgs1 to interact with dsDNA; in contrast, RECQ5 is not known to participate in either of these reactions.

Srs2 and Sgs1 can efficiently strip the Rad51 from ssDNA but are unable to dismantle Dmc1 filaments (41,62). Here, we find that RECQ5 can dismantle the DMC1 nucleofilament, but the processivity of RECQ5 on DMC1 filaments is significantly reduced compared to reactions with RAD51. This seems to correlate to the strength of interaction with individual proteins given that DMC1 and RPA showed very weak or no binding to RECQ5 whereas RAD51 protein does bind to RECQ5. Accordingly, RECQ5 also displays a lower inhibitory effect on DMC1 in a strand exchange reaction, supporting the preference of RECQ5 for clearing RAD51 over DMC1 nucleofilament. Nevertheless, the role of RECQ5 during meiosis is supported by observation that RECQ5 is strongly expressed in testis (72), so further investigation into RECQ5 function in meiosis is required.

Interestingly, although human BLM is considered an antirecombinase (3,34,73), we see no evidence that it can productively interact with either RPA–ssDNA or RAD51–ssDNA in our single molecule assays (35). It is important to recognize that BLM can only act upon the inactive ADP-bound form of the RAD51 filament (3,34), which is itself highly unstable (36,47,74), and BLM is unable to act upon the active ATP-bound form of the RAD51 filament (3,34,35). Indeed, we find that RPA and the active, ATP-bound form of RAD51 both block BLM interactions with ssDNA (35). In contrast, we do find that BLM exhibits robust dsDNA binding and unwinding activity (35), as previously reported (70,71). Thus, RECQ5 and BLM appear to have very different substrate specificities, perhaps reflecting unique roles in DNA repair.

### Division of labor among human RECQ helicases

WRN, BLM, RECQ1, RECQ4 and RECQ5 contribute widely to genome integrity through their involvement in a range of DNA repair pathways (1–7,9). Their biological importance is highlighted by the fact that mutations in three of these proteins are associated with genome instability diseases, namely Werner, Bloom and Rothmund–Thomson syndromes. However, it is not yet fully clear how these five helicases may be differentially regulated. They can interact with some shared protein partners and they may also have some functional overlap, making it difficult to fully appreciate their putative roles in the maintenance of genome integrity (1–7,9). Thus, a crucial issue in the field is to help define how these RecQ helicases are integrated into human DNA metabolism. It is possible that some of their *in vivo* functional characteristics may be defined at the level of substrate specificity. For instance, we can readily detect human RECQ5 disruption of RAD51 and DMC1 filaments and we can also see extensive translocation of RECQ5 on RPA–ssDNA. However, we can find no evidence for RECQ5 translocation or helicases activity on dsDNA molecules, and only limited evidence for unwinding when RECQ5 is specifically targeted to an DNA end, even though RECQ5 can bind to dsDNA and dsDNA supports RECQ5 DNA-dependent ATP hydrolysis activity. These observations suggest the possibility that RECQ5 activities may be restricted to ssDNA–containing reaction intermediates. As indicated above, these findings for RECQ5 are in striking contrast to our results with BLM helicase, which we find readily unwinds naked dsDNA but exhibits no binding or translocation activity on either RPA–ssDNA, RAD51–ssDNA or even RAD51-bound dsDNA substrates (35). In addition, BLM readily unwinds thousands of tens of kilobases of dsDNA when targeted to the DNA ends (C.X. and E.C.G., in preparation). Thus, BLM may be restricted to interactions with dsDNA-containing reaction intermediates. We speculate that the differential behaviors of RECQ5 and BLM towards ssDNA and dsDNA substrates may reflect a division of labor for these two human RecQ helicases with respect to substrate specificity during interactions with DNA replication or DNA repair intermediates.

The differences in substrate specificity for RECQ5 and BLM may arise from the differences in protein domain structure for these two RecQ helicases. For instance, the RecQ C-terminal (RCQ) domain is present in most RecQ helicases, including BLM and Sgs1, but this domain is truncated in RECQ5 (1,4,5,31). The RCQ domain of BLM contains a wing-helix (WH) motif that has been implicated in dsDNA binding and binding to dsDNA/ssDNA junctions, but this WH motif is absent from RECQ5, which may be anticipated to influence its interactions with DNA. Similarly, many RecQ helicases have a helicase and RNAaseD C-terminal (HRDC) domain (1,4,5,31). Interestingly, this HRDC domain is present in both BLM and WRN, as well as yeast Sgs1, but it is absent from RECQ1, RECQ4 and RECQ5 (1,4,5,31). The function of the RECQ helicase HRDC domain remains unknown, but the isolated HRDC domain from yeast Sgs1 can bind to dsDNA and the HRDC domain appears to play a role in the recruitment of WRN and BLM helicase to sites of DNA damage, so it may contribute somehow to substrate binding specificity (75–78). Their regulation by posttranslational modifications and specific protein–protein interactions might also play a key role. Indeed, RECQ helicases have been reported to exist in variety of possible subcomplexes (Croteau *et al.* 2014), linking them to DNA repair, replication, recombination and transcription. In moving forward, it will be important to examine and compare the mechanistic attributes of the other human RecQ helicase family members and it will also be important to fully define how the various protein domains and interaction factors contribute to the properties of these enzymes.

## Supplementary Material

gkaa1184_Supplemental_File
