# Supplementary material for: Single-molecule visualization of human RECQ5 interactions with single-stranded DNA recombination intermediates
Source: Nucleic Acids Res. 2020 Dec 17;49(1):285–305. doi: 10.1093/nar/gkaa1184 (PMC7797033; doi:10.1093/nar/gkaa1184)
Supplement: gkaa1184_Supplemental_File [file gkaa1184_supplemental_file.pdf]

SUPPLEMENTAL INFORMATION FOR:

Single–molecule visualization of human RECQ5 interactions with single–stranded DNA recombination intermediates

Chaoyou Xue[1‡], Lucia Molnarova[2‡], Justin B. Steinfeld[1], Weixing Zhao[5], Chujian Ma[1], Mario Spirek[2], Kyle Kaniecki[1], Youngho Kwon[5], Ondrej Beláň[6], Katerina Krejci[2,3], Simon J. Boulton[6], Patrick Sung[5], Eric C. Greene[1†] and Lumir Krejci[2,3,4†]

[1]Department of Biochemistry & Molecular Biophysics, Columbia University, New York, NY, 10032
[2]Department of Biology, Masaryk University, Brno 62500, Czech Republic
[3]International Clinical Research Center, St. Anne's University Hospital Brno, Brno 65691, Czech Republic
[4]National Centre for Biomolecular Research, Masaryk, Brno 62500, Czech Republic
[5]Department of Biochemistry and Structural Biology, University of Texas Health Science Center at San Antonio, Texas 78229
[6]DSB Repair Metabolism Lab, The Francis Crick Institute, Midland Road, London NW1 1AT, UK

[†]To whom correspondence should be addressed: ecg2108@cumc.columbia.edu and lkrejci@chemi.muni.cz
[‡] These authors made equal contributions

SUPPLEMENTAL FIGURE LEGENDS

Table S1. Summary of all initial RECQ5 ATP hydrolysis rates for all tested reaction conditions. This table shows the initial ATPase rate for RECQ5, RECQ5–F666A, and GFP–RECQ5 under each of the indicated reaction conditions. Reported values represent the mean and standard deviation of three different experiments.

Figure S1. Comparison of ATPase activity levels for RECQ5 and GFP–RECQ5 under different reaction conditions. (A) ATPase activity levels for RECQ5 and GFP–RECQ5 with either ssDNA or dsDNA, as indicated. **(B)** ATPase activity levels for RECQ5 and GFP–RECQ5 with ssDNA in the presence of various concentrations of RPA, as indicated. **(C)** ATPase activity levels for RECQ5 and GFP–RECQ5 with ssDNA in the presence of various concentrations of RAD51, as indicated. All data in this figure represent the 15–minute time points take from Figure 1B–F; individual data points represent the results of three independent experiments, bar heights represent the mean of the three experiments and the error bars represent the standard deviation of the three experiments; P values are provided for comparison of different data sets.

Figure S2. Summary of RECQ5 translocation characteristics under various reaction conditions. (A) Comparison of RECQ5 translocation velocities. Error bars represent 95% confidence intervals obtained through Gaussian fits. **(B)** Comparison of RECQ5 processivity values. Error bars represent 95% confidence intervals obtained through bootstrap analysis. Statistical analysis of the data sets were conducted by using a Student's T–test for comparison of the velocity and processivity results from the different reaction conditions to assays conducted with unlabeled RECQ5 and unlabeled RAD51 using RPA–GFP as a readout for translocation activity (corresponding to the second data columns in each panel).

Figure S3. Photobleaching and AFM analysis of RECQ5. (A) Kymograph showing photobleaching of GFP–RECQ5 (green) under constant laser illumination conditions. The location of the GFP–RECQ5 was first confirmed under shuttered illumination conditions (as indicated), and then switched to continuous illumination to induce photobleaching. **(B)** Example of GFP–RECQ5 photobleaching steps. **(C)** Distribution of observed photobleaching steps; note, examples categorized as having ≥4 steps were too bright to definitely ascribe to a specific number of photobleaching steps. **(D)** Volumetric analysis of AFM images (see inset) of RECQ5 alone or **(E)** RECQ5 in the presence of a 49–nt ssDNA fragment. The frequency of occurrence is shown as the percentage of the various signals of a defined molecular mass. **(F)** Example of an AFM image for RECQ5 in the absence of DNA. **(G)** Example of an AFM image for RECQ5 in the presence of DNA.

Figure S4. BLI analysis of RECQ5–mediated disruption of RAD51–K133R, RAD51–K133A and RAD51–I287T nucleofilaments. (A) BLI traces for RECQ5–mediated interferometry change on RAD51–WT and RAD51–K133R nucleofilaments. The data from the time period where BRC3 was added to the RAD51 filament in the absence or presence of RECQ5, were normalized to the starting point. The % of optical thickness change was then plotted as a function of time for each nucleoprotein filament. **(B)** Destabilization of RAD51–WT and RAD51–K133R nucleoprotein filaments by BRC3 repeats in the absence of RECQ5. BLI traces for BRC3–mediated interferometry change on RAD51–WT and RAD51–K133R nucleofilaments. The data from the time period where BLI buffer was added to the RAD51 filament in the presence of BRC3 were normalized to the starting point. The % of optical thickness change was then plotted as a function of time for RAD51–WT (black) and RAD51–K133R (red) nucleoprotein filament. **(C)** BLI traces for RECQ5–mediated interferometry change on RAD51–WT and RAD51–K133A. The data from the

time period where BRC3 was added to the RAD51 filament in the absence or presence of RECQ5, were normalized to the starting point. The % of optical thickness change was then plotted as a function of time for each nucleoprotein filament. **(D)** BLI traces for RECQ5–mediated interferometry change on RAD51–WT and RAD51–I287T. The data from the time period where BRC3 was added to the RAD51 filament in the absence or presence of RECQ5, were normalized to the starting point. The % of optical thickness change was then plotted as a function of time for each nucleoprotein filament. **(E)** BLI analysis of RAD51 binding to surface immobilized GST–BRC3. The indicated RAD51 variants (1 μM each) were bound to anti-GST biosensors coated with GST–BRC3 (association phase) and after 120 seconds the biosensor was flushed with protein–free buffer to initiate RAD51 dissociation (dissociation phase).

Figure S5. Assembly and disassembly kinetics for DMC1. (A) Kymographs showing the assembly and disassembly of DMC1 filaments on single ssDNA molecules in the presence of 0.5 nM RPA–GFP (green). Assembly reactions were initiated by injecting 2 μM DMC1 together with 2.5 mM ATP and 1.5 mM Ca^{2+} into sample chambers containing ssDNA molecules coated with RPA–GFP while monitoring the loss of RPA–GFP signal. Disassembly reactions were initiated by flushing ATP and Ca^{2+} from the sample chambers while monitoring the increase in ssDNA–bound RPA–GFP signal. **(B)** Graphs showing the normalized RPA–GFP signal intensity integrated over entire ssDNA molecules during the assembly of the DMC1 filaments. Shaded error bars represent 68% confidence intervals of indicated ssDNA molecules. **(C)** Graphs showing the normalized RPA–GFP signal intensity integrated over entire ssDNA molecules during DMC1 filament disassembly. Shaded error bars represent 68% confidence intervals of indicated ssDNA molecules.

Figure S6. BLI analysis of RAD51, DMC1 or RPA interaction with RECQ5. Increasing concentrations (as indicated) of either **(A)** RAD51, **(B)** DMC1 or **(C)** RPA were bound to Protein A biosensors coated by RECQ5 (association phase), and after 120 seconds flushed with protein–free buffer to induce dissociation. **(D)** Plot of observed end–points of optical thickness for corresponding interactions with RECQ5 as function of protein concentration.

Table S1

Reaction conditions	Initial ATP hydrolysis rate (μM/s)
RECQ5 + dsDNA	2.00 ± 0.0056
GFP–RECQ5 + dsDNA	1.92 ± 0.015
RECQ5 + ssDNA + 0 μM RAD51	2.92 ± 0.00060
RECQ5 + ssDNA + 0.5 μM RAD51	2.54 ± 0.0058
RECQ5 + ssDNA + 1.0 μM RAD51	2.06 ± 0.0031
RECQ5 + ssDNA + 3.0 μM RAD51	1.79 ± 0.0024
RECQ5–F666A+ ssDNA + 0 μM RAD51	2.52 ± 0.0069
RECQ5–F666A + ssDNA + 0.5 μM RAD51	2.14 ± 0.0049
RECQ5–F666A + ssDNA + 1.0 μM RAD51	1.76+ 0.0057
RECQ5–F666A + ssDNA + 3.0 μM RAD51	1.20 ± 0.0021
GFP–RECQ5 + ssDNA + 0 μM RAD51	2.57 ± 0.00071
GFP–RECQ5 + ssDNA + 0.5 μM RAD51	2.12 ± 0.0044
GFP–RECQ5 + ssDNA + 1.0 μM RAD51	1.89 ± 0.0025
GFP–RECQ5 + ssDNA + 3.0 μM RAD51	1.44 ± 0.0032
RECQ5 + ssDNA + 0 μM RPA	1.128 ± 0.079
RECQ5 + ssDNA + 0.05 μM RPA	1.26 ± 0.065
RECQ5 + ssDNA + 0.2 μM RPA	1.61 ± 0.059
RECQ5 + ssDNA + 1.0 μM RPA	1.06 ± 0.030
RECQ5–F666 + ssDNA + 0 μM RPA	1.42 ± 0.18
RECQ5–F666 + ssDNA + 0.05 μM RPA	1.60 ± 0.11
RECQ5–F666 + ssDNA + 0.2 μM RPA	1.70 ± 0.067
RECQ5–F666 + ssDNA + 1.0 μM RPA	0.91 ± 0.096
GFP–RECQ5 + ssDNA + 0 μM RPA	1.30 ± 0.026
GFP–RECQ5 + ssDNA + 0.05 μM RPA	2.04 ± 0.35
GFP–RECQ5 + ssDNA + 0.2 μM RPA	1.44 ± 0.12
GFP–RECQ5 + ssDNA + 1.0 μM RPA	1.07 ± 0.077

Figure S1

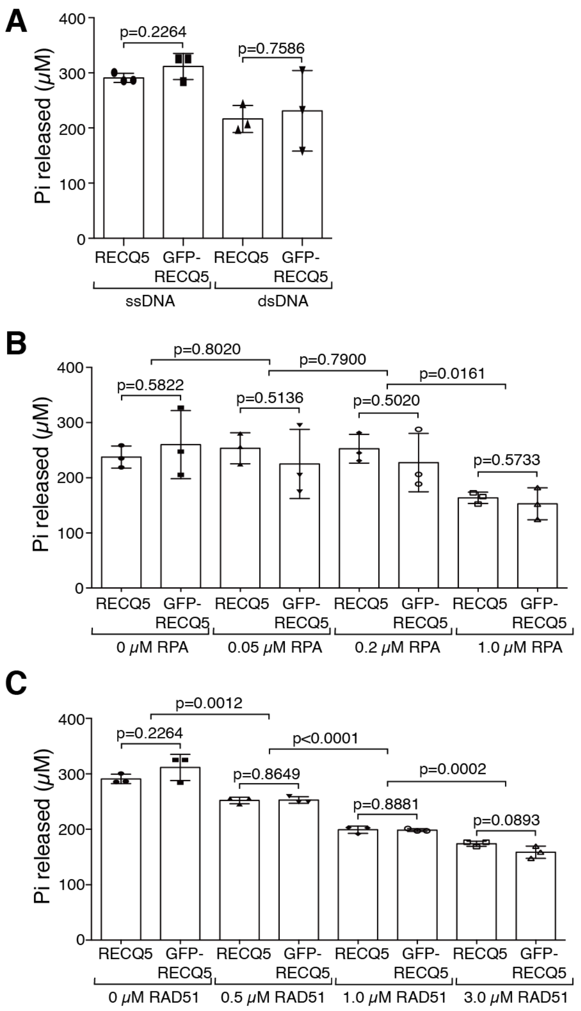
A
Pi released (μM)
p=0.2264
p=0.7586
RECQ5
GFP-RECQ5
ssDNA
dsDNA
B
p=0.8020
p=0.7900
p=0.0161
p=0.5822
p=0.5136
p=0.5020
p=0.5733
0 μM RPA
0.05 μM RPA
0.2 μM RPA
1.0 μM RPA
C
p=0.0012
p<0.0001
p=0.0002
p=0.2264
p=0.8649
p=0.8881
p=0.0893
0 μM RAD51
0.5 μM RAD51
1.0 μM RAD51
3.0 μM RAD51

Figure S2

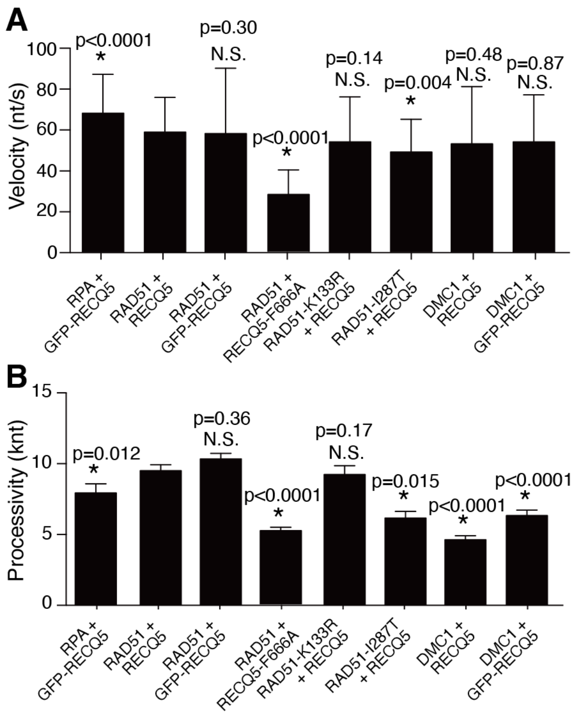
A
Velocity (nt/s)
p<0.0001
p=0.30
N.S.
p<0.0001
p=0.14
N.S.
p=0.004
p=0.48
N.S.
p=0.87
N.S.
RPA + GFP-RECQ5
RAD51 + RECQ5
RAD51 + GFP-RECQ5
RAD51 + RECQ5-F666A
RAD51-K133R + RECQ5
RAD51-I287T + RECQ5
DMC1 + RECQ5
DMC1 + GFP-RECQ5
B
Processivity (knt)
p=0.012
p=0.36
N.S.
p<0.0001
p=0.17
N.S.
p=0.015
p<0.0001
p<0.0001

Figure S3

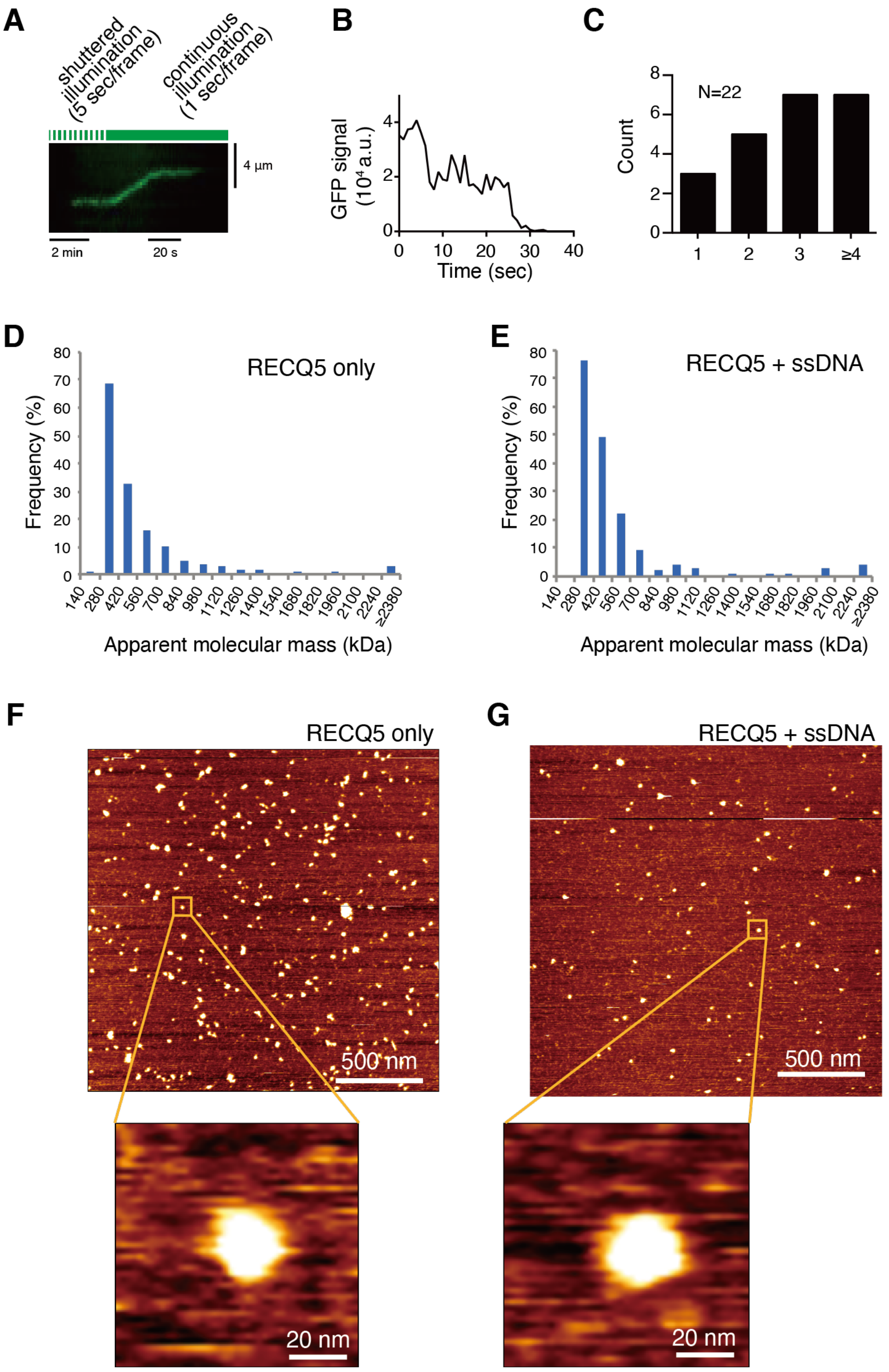
A
shuttered illumination (5 sec/frame)
continuous illumination (1 sec/frame)
4 µm
2 min
20 s
B
GFP signal (10^4 a.u.)
Time (sec)
C
N=22
Count
D
RECQ5 only
Frequency (%)
Apparent molecular mass (kDa)
E
RECQ5 + ssDNA
Frequency (%)
Apparent molecular mass (kDa)
F
RECQ5 only
500 nm
20 nm
G
RECQ5 + ssDNA
500 nm
20 nm

Figure S4

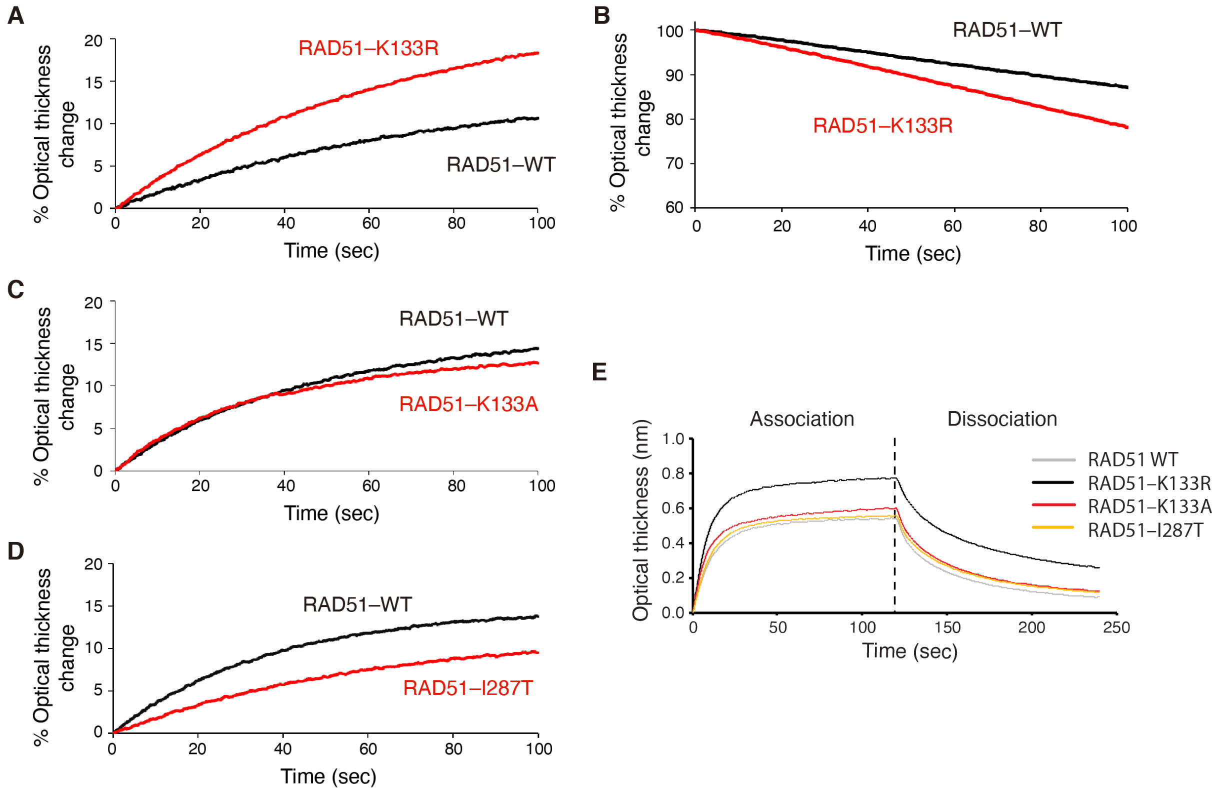

A
% Optical thickness change
RAD51–K133R
RAD51–WT
Time (sec)
B
% Optical thickness change
RAD51–WT
RAD51–K133R
Time (sec)
C
% Optical thickness change
RAD51–WT
RAD51–K133A
Time (sec)
D
% Optical thickness change
RAD51–WT
RAD51–I287T
Time (sec)
E
Association
Dissociation
Optical thickness (nm)
RAD51 WT
RAD51–K133R
RAD51–K133A
RAD51–I287T
Time (sec)

Figure S5

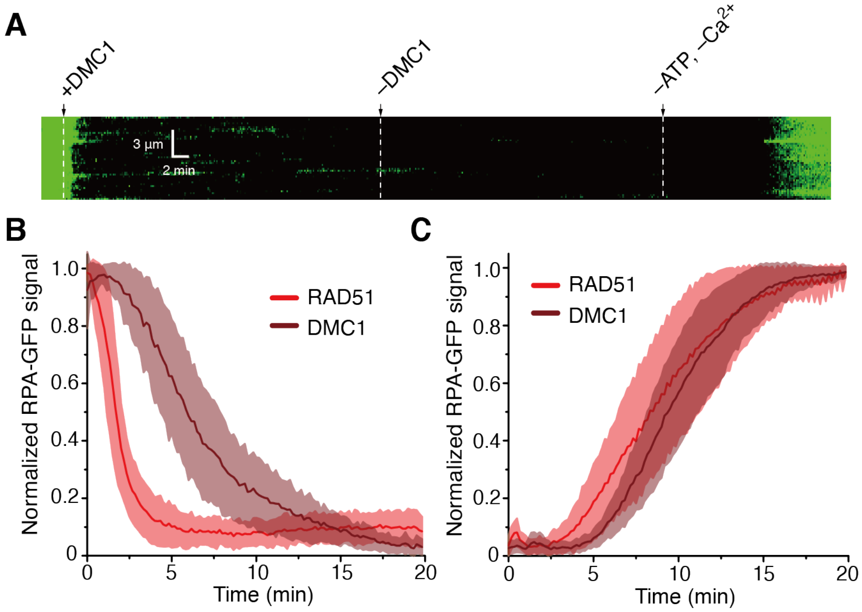
A
+DMC1
–DMC1
–ATP, –Ca2+
3 µm
2 min
B
C
Normalized RPA-GFP signal
RAD51
DMC1
Time (min)

Figure S6

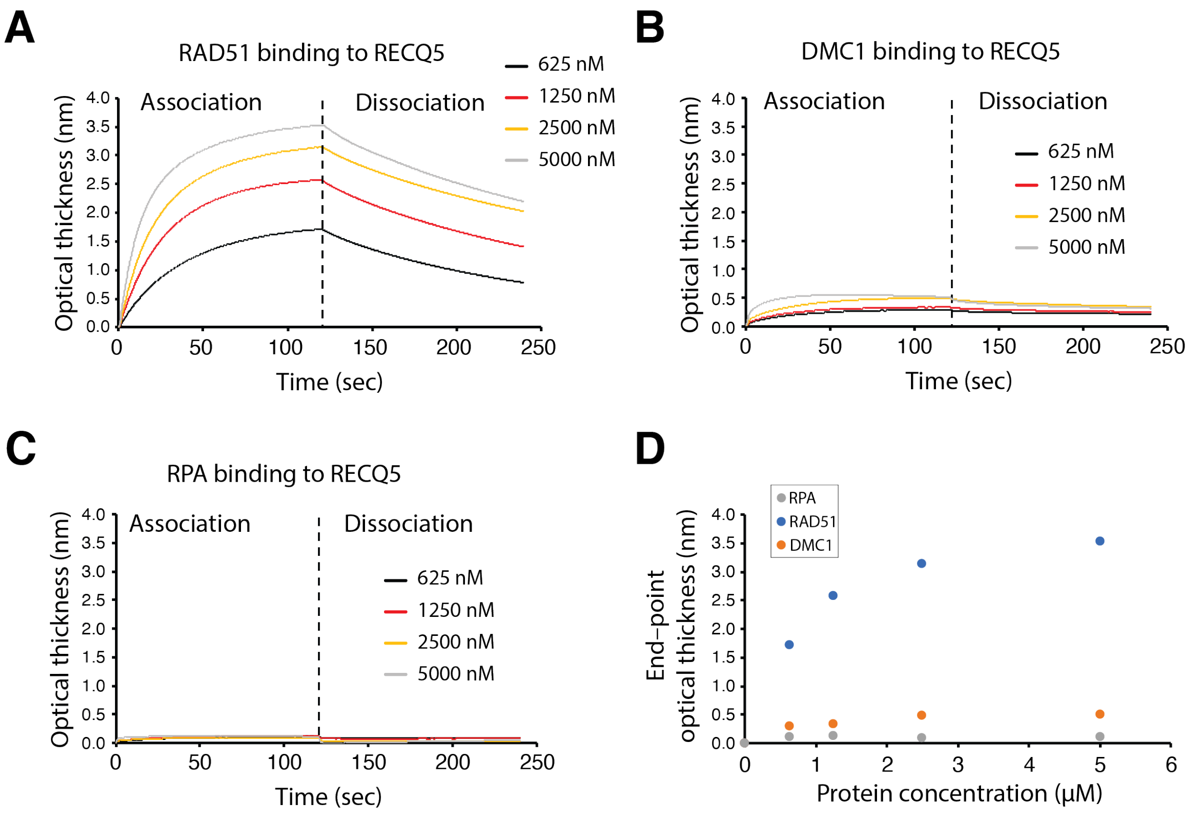
A
RAD51 binding to RECQ5
Association
Dissociation
625 nM
1250 nM
2500 nM
5000 nM
Optical thickness (nm)
Time (sec)
B
DMC1 binding to RECQ5
Association
Dissociation
625 nM
1250 nM
2500 nM
5000 nM
Optical thickness (nm)
Time (sec)
C
RPA binding to RECQ5
Association
Dissociation
625 nM
1250 nM
2500 nM
5000 nM
Optical thickness (nm)
Time (sec)
D
RPA
RAD51
DMC1
End-point optical thickness (nm)
Protein concentration (μM)
